# Human broadly neutralizing influenza B virus antibodies recognizing hemagglutinin computationally optimized broadly reactive antigens

**DOI:** 10.3389/fimmu.2026.1747235

**Published:** 2026-03-25

**Authors:** Yailin Campos Mota, John V. Dzimianski, Mariana Lopez, Robert A. Richardson, Ted M. Ross, Ian J.A. Kunkel, Sara M. O’Rourke, Vitor Hugo Balasco Serrão, Rebecca M. DuBois, Giuseppe A. Sautto

**Affiliations:** 1 Florida Research and Innovation Center, Cleveland Clinic, Port Saint Lucie, FL, United States; 2 Department of Biomolecular Engineering, University of California Santa Cruz, Santa Cruz, CA, United States; 3 Center for Vaccines and Immunology, University of Georgia, Athens, GA, United States; 4 Department of Infectious Diseases, University of Georgia, Athens, GA, United States; 5 Department of Infection Biology, Cleveland Clinic Research, Cleveland, OH, United States; 6 Department of Chemistry and Biochemistry, University of California Santa Cruz, Santa Cruz, CA, United States; 7 Biomolecular cryo-EM Facility, University of California Santa Cruz, Santa Cruz, CA, United States

**Keywords:** broadly neutralizing antibodies (bnAbs), hemagglutinin (HA), influenza B virus (IBV), monoclonal antibodies (mAbs), quadrivalent influenza vaccine (QIV)

## Abstract

**Introduction:**

Influenza B viruses (IBVs) are responsible for severe disease and death, similarly to influenza A viruses (IAVs), with a higher number of infections happening in children and the elderly. Despite the inclusion of an IBV component in the seasonal influenza vaccine, rates of vaccine effectiveness (VE) are still variable and low in many previous seasons.

**Methods:**

In this work, longitudinal profiling of IBV hemagglutinin (HA)-specific B-cell responses was described following influenza vaccination in 15 vaccinated participants over four consecutive influenza seasons. These individuals belonged to different age groups and monoclonal antibodies (mAbs) showing broad binding and functional antibody profiles were isolated from the plasmablasts of one individual.

**Results:**

These individuals possessed different breadths and magnitudes of antibody responses to a panel of IBV historical and more recent vaccine strains. In particular, young adults (age 23–33) showed a higher magnitude and breadth of antibody response compared to middle-aged (age 55–58) and elderly (age 65–77) participants who instead showed a lower albeit detectable antibody response. Interestingly, one of the isolated mAbs, mAb #46, had the broadest response with a broad binding and potent functional activity against historical and recent IBV strains spanning both Victoria and Yamagata lineages and including binding to IBV computationally optimized broadly reactive antigen (COBRA) HAs. Importantly, mAb #46 administration, either therapeutically or prophylactically, fully protected IBV-challenged mice. Structural characterization of the mAb #46–HA complex by cryo-electron microscopy single-particle analysis revealed that mAb #46 targets a conserved epitope within the HA receptor binding site.

**Discussion:**

This study highlights the presence of broadly neutralizing antibodies in the human repertoire that may be recalled by vaccination with COBRA HA, although this hypothesis will be confirmed in upcoming clinical trials.

## 1 Introduction

Similar to other respiratory infectious agents, influenza viruses cause a meaningful societal and economic burden every year. Seasonal influenza types A (IAV) and B (IBV) represent the primary circulating strains responsible for significant morbidity within the human population. IBVs lack an animal reservoir compared to IAVs, and this is one of the reasons for their lower outbreak incidence and absence of antigenic shift as observed in IAVs. However, before the SARS-CoV-2 pandemic, seasonal IBV epidemics were characterized by increasing unpredictability and heightened virulence, particularly among vulnerable cohorts. Along with developing better countermeasures for IAVs, a need for improved prevention and therapeutic strategies for IBVs has to be considered.

Before the COVID-19 pandemic, up to 650,000 deaths worldwide were directly attributed to influenza virus infection and ~11% of the U.S. population was typically infected each year according to the Centers for Disease Control and Prevention (CDC) (1).

The main virus surface glycoprotein hemagglutinin (HA) is the main target of current standard of care (SOC) vaccines, as well as under development influenza vaccines. In fact, HA is the main target of the antibody response against influenza (2).

IBVs are separated into two lineages, B/Victoria-like and B/Yamagata-like, based on HA antigenic differences and named after the respective reference strains, B/Victoria/2/1987 and B/Yamagata/16/1988 (Yam/88) (3). Both lineages have been co-circulating globally for over four decades. However, since April 2020, influenza B/Yamagata viruses have not been consistently detected in global surveillance systems. For this reason, B/Yamagata-like strains are not included in the seasonal influenza vaccine (4).

Although licensed vaccines are available, their efficacy is often undermined by the continuous evolution of circulating strains. High genetic diversity remains the primary barrier to developing a single formulation capable of broad protection against divergent IBV strains. This is largely driven by antigenic drift, which is the accumulation of point mutations within HA that enables the virus to evade host memory immune recognition. Driven by fitness pressure, these mutations preferentially occur in major antigenic regions, allowing the virus to bypass neutralizing antibodies. These sites are found in the HA1 domain of the protein and consist of the 120-loop, 150-loop, 160-loop, and 190-helix (5). While the immune system can develop neutralizing antibodies against other HA regions, these are typically acquired only with age and repeated IBV exposure. To address this, innovative vaccine design strategies are being developed to elicit broader, cross-reactive immune responses capable of neutralizing genetically diverse strains. These advancements are particularly vital given recent evidence emphasizing the severe impact of IBV on high-risk groups, most notably children and the elderly. Additionally, the 2019–2020 influenza season saw the emergence of a new clade of IBV, which was associated with a disparate circulation pattern compared to typical influenza seasons (6). These findings underscore the substantial disease burden imposed by IBVs, necessitating a level of dedicated research parity with that traditionally afforded to IAVs.

Because of the severity of disease and the probable continued circulation, it is important to shed light on the immune response elicited by the IBV vaccine to develop novel broadly effective immunotherapeutic countermeasures and to help in novel vaccine design and development.

Among the different strategies for developing next-generation influenza vaccines, computationally optimized broadly reactive antigens (COBRAs) elicit broadly neutralizing antibody responses for both IAVs and IBVs of different subtypes and lineages, respectively. COBRA vaccines have been described for IAV H1, H2, H3, H5, and H7 and IBV HAs as well as for IAV neuraminidase (N1), using a variety of different vaccine platforms (7–11). These vaccines have shown promise in a variety of pre-clinical animal models of influenza infection while also showing effectiveness in pre-immune models of vaccination (12–15). Overall, COBRA HA vaccines induce a broader protection than commercially available vaccines or wild-type comparator strains. Understanding the pre-existing human antibody response to these immunogens is fundamental for their further refinement and characterizing the antibody determinants that can be recalled in the context of upcoming clinical trials with next-generation influenza vaccines.

In this study, we have longitudinally in-depth profiled the antibody response of individuals from different age groups that received the SOC quadrivalent influenza vaccine (QIV). After having assessed their serological and memory B (B_mem_) cell responses, the binding and functional activity of monoclonal antibodies (mAbs) derived from one of these individuals was characterized, and mAbs endowed with a broad binding and functional profile, spanning the two IBV lineages, as well as IBV COBRA HA, were identified.

These findings suggest that IBV HA-specific broadly reactive and neutralizing antibodies recognizing COBRA HA exist in the human antibody repertoire and may potentially be recalled upon vaccination by next-generation influenza vaccines like COBRA.

## 2 Materials and methods

### 2.1 Subjects and vaccine

From 2013 to 2016, eligible participants aged 17 and older who had not yet received the seasonal influenza vaccine were enrolled starting in September of each year. These volunteers were recruited in the United States from two sites that included medical facilities in Pittsburgh (Pennsylvania) and Stuart (Florida) (16, 17). Exclusion criteria consisted of documented contraindications such as Guillain–Barré syndrome, dementia or Alzheimer’s disease, and egg allergies. Additionally, individuals with a life expectancy of less than 2 years, immunocompromising conditions, or concurrent enrollment in other influenza trials were excluded. While low community transmission precluded active viral monitoring, participants were screened for influenza-like symptoms at each visit; symptomatic individuals were removed from the study. In accordance with World Health Organization (WHO) recommendations for Northern Hemisphere influenza seasons, all vaccinations and sample collections took place between September and March from 2013 to 2017. Subjects were vaccinated with the standard dose or high-dose (if 65+ years old) split-virion inactivated influenza vaccine (IIV) version of licensed Fluzone (Sanofi Pasteur, Swiftwater, PA, USA). Participants received either the intradermal (available in the 2013–2014 season only) or the intramuscular (available in all the seasons) formulation of the IIV. The quadrivalent IIV formulation consisted of four strains of the influenza virus according to the WHO recommendations for the 2013–2017 seasons (**Table 1**).

**Table 1:** IBV composition and formulation of the seasonal inactivated quadrivalent influenza vaccine (QIV) across the different seasons analyzed in this study.

QIV season	QIV formulation and number of participants	IBV component
2013–2014	*n* = 8 SD; *n* = 6 ID; *n* = 1 HD	B/Brisbane/60/2008 (V) B/Massachusetts/2/2012 (Y)
2014–2015	*n* = 12 SD; *n* = 2 HD	
2015–2016	*n* = 11 SD; *n* = 2 HD	B/Brisbane/60/2008 (V) B/Phuket/3073/2013 (Y)
2016–2017	8 SD; 4 HD	

V, Victoria lineage; Y, Yamagata lineage; SD, standard dose; ID, intradermal; HD, high dose. For the QIV formulation, the number of participants receiving the corresponding formulation is reported.

Fifteen eligible participants (23–77 years old) showing a seroconversion, defined as a fourfold increase in hemagglutination inhibition (HAI) titer compared to baseline resulting in a titer of ≥1:40, as per the WHO and European Committee for Medicinal Products to evaluate influenza vaccines, were selected for this study (**Table 2**). While most subjects completed the longitudinal study across all seasons, participation among the young adult group declined over time: five individuals participated in the 2013–2014 season, four in 2014–2015, three in 2015–2016, and two in 2016–2017.

**Table 2:** Human cohort composition in terms of age groups, gender, and ethnicity of vaccinees.

Seasons 2013–2016			Age groups (2013)		
Subjects		Total	23–33 (Y)	55–58 (M)	65–77 (E)
		15	5	6	4
Gender	♀	11 (73%)	4 (36.4%)	4 (36.4%)	3 (27.2%)
	♂	4 (27%)	1 (25%)	2 (50%)	1 (25%)
Ethnicity	White/Caucasian	6 (40%)	3 (20%)	2 (13%)	1 (7%)
	Black/AA	7 (47%)	0	4 (27%)	3 (20%)
	Hispanic/Latino	2 (13%)	2 (13%)	0	0

(Y): young adults; (M): middle aged; (E): elderly.

**Figure 1:**
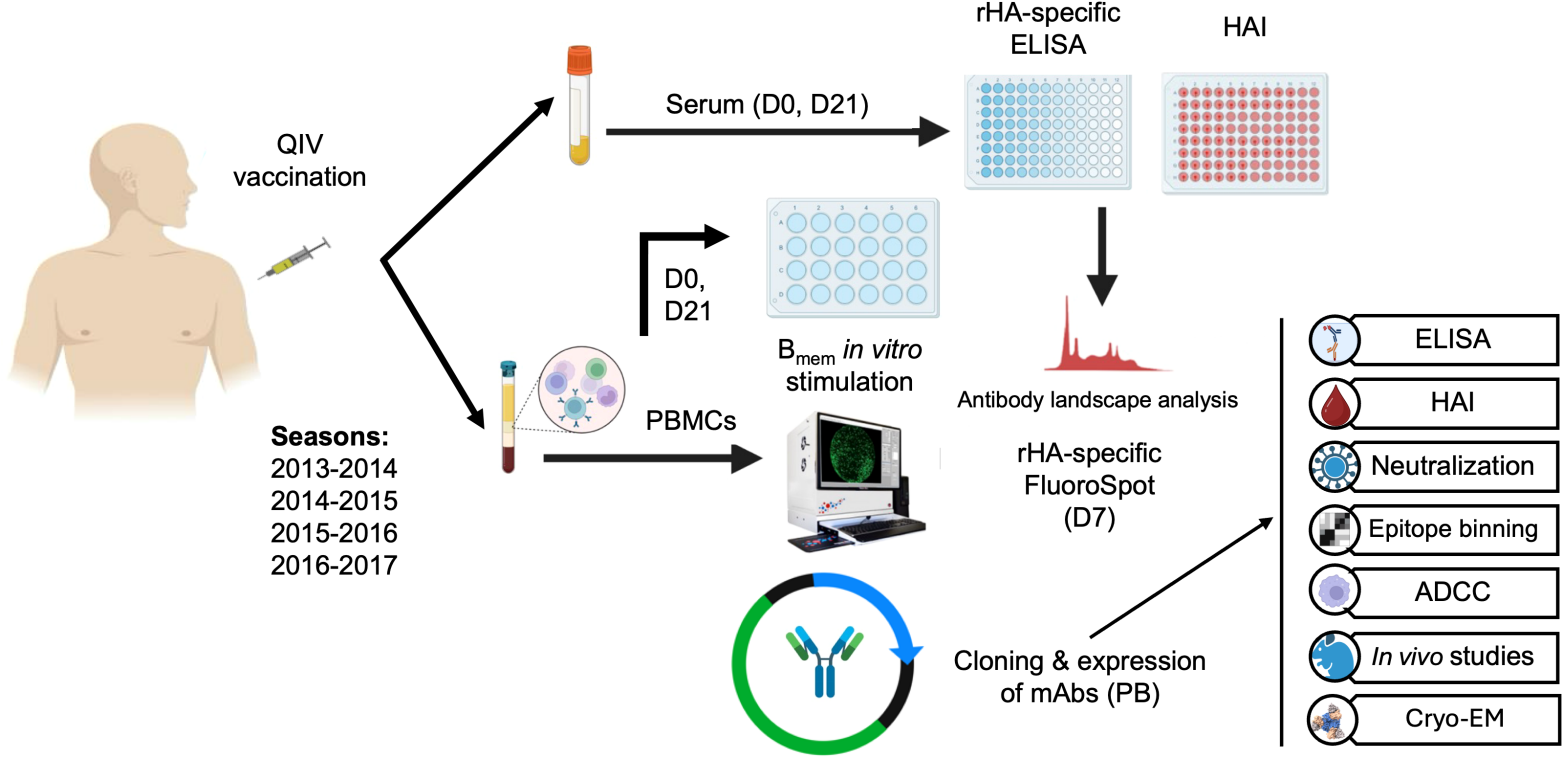
Schematic representation of the study. Following consecutive administration of seasonal inactivated QIV, serum and PBMCs were collected from human subjects belonging to different age groups at baseline (day 0, D0), day 7 (D7),and day 21 (D21). ELISA and HAI assays were performed with collected sera using a panel of historical and recent recombinant HA protein strains and IBVs, respectively, to assess the breadth of recognition and HAI of participants at the polyclonal level. D0 and D21 PBMC samples were *in vitro* stimulated and used to assess the magnitude of IBV HA-specific secreted IgG. D7 PBMC samples were used to assess the magnitude and frequency of IBV HA-specific IgG, IgM, and IgA antibody secreting cells (ASCs). D7 samples were used to isolate IBV HA-specific plasmablasts (PBs), and antibodies of interest were cloned, recombinantly expressed, and characterized for their binding and functional profile through *in vitro* and *in vivo* studies.

Whole blood (70–90 mL) was collected from each subject at baseline at the time of vaccination (D0) and 7–9 days (D7) and 21–28 days (D21) after vaccination to facilitate subject recruitment and compliance. Blood samples were collected for serum and peripheral blood mononuclear cells (PBMCs) (**Figure 1**). For the isolation of PBMCs, blood was collected in Vacutainer CPT tubes (catalog no. 362753, BD Biosciences, Franklin Lakes, NJ, USA) at D0, D7, and D21. Samples were processed within 6 h of collection and cryopreserved in the vapor phase of liquid nitrogen for subsequent analysis. Corresponding serum was collected using Vacutainer SST tubes (BD Biosciences, catalog no. 367989). Samples were maintained at 4 °C for 24–48 h until processing, at which point they were separated and aliquoted for long-term storage at –20 °C.

### 2.2 Ethics statement

The study procedures, informed consent, and data collection documents were reviewed and approved by the Western Institutional Review Board and the Institutional Review Boards of the University of Pittsburgh. Informed written consent was obtained from the enrolled participants of the study.

### 2.3 Cell lines

MDCK (Madin–Darby Canine Kidney) (catalog no. CCL-34, ATCC, Manassas, VA, USA) cells were cultured in Dulbecco’s Modified Eagle’s Medium (DMEM) supplemented with 5 U/mL penicillin–5 μg/mL streptomycin (catalog no. 15070063), bovine serum albumin (BSA) fraction V 7.5% solution (catalog no. 15260037), 25 mM HEPES buffer (catalog no. 15630080), and 10% heat-inactivated fetal bovine serum (FBS; catalog no. S11550, R&D Systems, Minneapolis, MN, USA).

Stable transfected MDCK cells with the cDNA of human 2,6-sialtransferase (SIAT1) MDCK-SIAT1 (provided by the CDC, Atlanta, GA, USA) were maintained similarly to MDCK cells and supplemented with 1 mg/mL of geneticin (G418 sulfate). EXPI Human Embryonic Kidney (HEK) 293F (Expi293F) cells (catalog no. A14527) were grown in Expi293 Expression Medium (catalog no. A1435101) and maintained according to the manufacturer’s instructions. All media and supplements except FBS were purchased from Thermo Fisher Scientific (Waltham, MA, USA).

### 2.4 Influenza viruses

Influenza virus isolates were obtained from the Influenza Reagents Resource (IRR), BEI Resources, or the CDC, or they were provided by Sanofi Pasteur and Virapur LLC (San Diego, CA, USA). Viruses were passaged once in the same growth conditions as they were received, in 10-day-old embryonated, specific pathogen-free (SPF) chicken eggs (catalog no. 10100326, AVSBio, Norwich, CT, USA) per the protocol provided by the WHO (18).

For the pre-split lineage, the following viruses were used: B/Lee/1940 (Lee/40) and B/Singapore/1964 (Sing/64).

For the Victoria lineage, the following viruses were used: B/Hong Kong/330/2001 (HK/01), B/Malaysia/2506/2004 (MY/04), B/Victoria/304/2006 (Vic/06), B/Brisbane/60/2008 (Brisb/08), B/Colorado/06/2017 (CO/17), B/Washington/2/2019 (WA/19), and B/Austria/1359417/2021 (AU/21).

For the Yamagata lineage, the following viruses were used: B/Yamagata/16/1988, B/Harbin/7/1994 (Har/94), B/Memphis/20/1996 (Mem/96), B/Sichuan/379/1999 (Sich/99), B/Shanghai/361/2002 (SH/02), B/Florida/4/2006 (FL/06), B/Washington/2/2010 (WA/10), B/Wisconsin/1/2010 (WI/10), B/Texas/6/2011 (TX/11), B/Massachusetts/2/2012 (MA/12), and B/Phuket/3073/2013 (PH/13).

### 2.5 Recombinant HA proteins

All the recombinant HA (rHA) proteins described below were expressed in Expi293F cells and purified via a C-terminal histidine tag using HisTrap excel nickel-affinity chromatography columns (catalog no. 17371205, Cytiva, Marlborough, MA, USA) as previously described (19). Purified rHA proteins were dialyzed against phosphate buffered saline (PBS, catalog no. 21-040-CM, Corning, Corning, NY, USA), and total protein concentration was adjusted to ~1 mg/mL after bicinchoninic acid (BCA) assay (catalog no. 23227, Thermo Fisher Scientific) estimation. Purity was assessed by sodium dodecyl sulfate–polyacrylamide gel electrophoresis (SDS-PAGE) and Western blot (Thermo Fisher Scientific).

For binding assays, the full-length ectodomain of HA proteins was expressed and purified for the IBV COBRAs BC2, BC3, and BC17 as well as for recent and historical vaccine or wild-type (wt) components belonging to the Victoria (HK/01, Brisb/08, CO/17, WA/19, AU/21) or Yamagata lineage (SH/02 and PH/13).

Full-length ectodomains of IBV from Yamagata lineage strain B/Jilin/20/2003 (Jilin/03) (catalog no. NR-19242) and Victoria lineage strain B/Ohio/1/2005 (catalog no. NR-19243) were obtained through BEI Resources, National Institute of Allergy and Infectious Diseases (NIAID), a component of the U.S. National Institutes of Health (NIH).

Full-length ectodomains of IBV from Yamagata lineage strains FL/06 and WI/10 and Victoria lineage strain B/Malaysia/2506/2004 (MY/04) were obtained from Protein Sciences (Meriden, CT, USA).

rHA monomers (catalog no. IT-003-B19δlTMp) or HA1 (catalog no. IT-003-0019p) of MA/12 were purchased from Immune Technology Corp. (New York, NY, USA).

### 2.6 B-cell FluoroSpot assay

The number of total and HA-specific antibody-secreting cells (ASCs) was determined using the human immunoglobulin A/G/M (IgA/IgG/IgM) three-color FluoroSpot kit from CTL (Cellular Technology Ltd, Cleveland, OH, USA) based on the instructions provided by the manufacturer. Briefly, high-binding polyvinylidene fluoride (PVDF) filter plates were coated with anti-human Igκ/λ mixture (for total Ig plates) or 25 µg/mL of full-length rHA antigens from PH/13 or BC2 (for antigen-specific plates). PBMCs from the D7 time point following vaccination [for assessing the plasmablast (PB) response] were serially diluted threefold in B-cell medium [prepared as previously described (20)] starting at 5 × 10^4^ or 1 × 10^5^ cells, respectively, in duplicates. Following a 16-h incubation at 37°C in 5% CO_2_, the plates were washed and treated with a mixture of secondary IgA/IgG/IgM detection antibodies for 2 h in the dark. Subsequently, the plates were washed and incubated with a tertiary antibody solution containing SA-CTL-Red™ and Anti-Hapten CTL-Yellow™ for 1 h at room temperature (RT). After a final wash and decanting, the membranes were dried thoroughly in the dark. Spots were scanned and quantified using an S6 Ultimate M2 ImmunoSpot reader and ImmunoSpot 7.0.28.5 Analyzer software (Cellular Technology Ltd).

### 2.7 *In vitro* stimulation of B cells

B_mem_ cells were *in vitro* stimulated for antibody secretion following previously described protocols (21).

In brief, PBMC samples were cultured (2 × 10^6^ viable cells/mL) in complete B-cell medium containing RPMI 1640 medium and 23.8 mM sodium bicarbonate (catalog no. R8758, Millipore Sigma) and supplemented with 10% FBS (R&D Systems), 25 mM HEPES (Thermo Fisher Scientific), 5 U/mL penicillin–5 μg/mL streptomycin (Thermo Fisher Scientific), 50 μM 2-mercaptoethanol (catalog no. M3148, Millipore Sigma), 1 mM sodium pyruvate (catalog no. 11360070, Thermo Fisher Scientific), essential amino acid solution (catalog no. 11130051, Thermo Fisher Scientific), nonessential amino acid solution (catalog no. 11140035, Thermo Fisher Scientific), 500 ng/mL R848 (catalog no. tlrl-r848-1, Invivogen), and 5 ng/mL rIL-2 (catalog no. BT-002, R&D Systems) for 7–9 days at 37 °C in 5% CO_2_ (21–24). Medium from conditioned supernatants was harvested and assayed for total and rHA-specific IgG content by enzyme-linked immunosorbent assay (ELISA) at a 1:20 dilution as described in the ELISA section.

### 2.8 Monoclonal antibody cloning, expression, and purification

Heavy (HC) and light (LC) chain of selected mAbs from participant #253 were synthesized and cloned as previously described in the pcDNA™3.4 TOPO vector (catalog no. A14697, Thermo Fisher Scientific) and expressed in HEK293F cells by GenScript (Piscataway, NJ, USA) (25). The expressed mAbs were purified from the conditioned supernatants of transfected cells and quantified, as previously described (26). Briefly, mAbs were purified from conditioned supernatants via affinity chromatography using HiTrap Protein G HP columns (Cytiva, catalog no. 17040501) on an ÄKTA Pure system (Cytiva). Antibodies were eluted with 0.1 M glycine (pH 2.5) and immediately neutralized with 200 μL of 1.5 M Tris (pH 8.8) (Millipore Sigma). Protein-rich fractions were pooled, buffer-exchanged into PBS containing 0.05% sodium azide, and concentrated using Amicon Ultra-15 Centrifugal Filter Units (catalog no. UFC905024, Millipore Sigma). Final mAb concentrations were determined using a Micro BCA Assay kit (catalog no. 23235, Thermo Fisher Scientific) against a human IgG standard (catalog no. 401114, Millipore Sigma), with purity confirmed via SDS-PAGE and Western blot (Thermo Fisher Scientific). All recombinant mAbs were successfully purified with yields ranging from 0.5 to 1 mg/mL of transfected culture.

### 2.9 Enzyme-linked immunosorbent assay

Immulon 4HBX ELISA plates (Thermo Fisher Scientific) were coated with 50 ng/well of rHA in PBS and incubated for 16 h at 4 °C in humidified chambers. Following incubation, plates were blocked with 2% BSA and 1% gelatin in PBS-T (0.05% Tween 20) for 2 h at 37 °C. To assess binding breadth, D0 and D21 serum samples (starting dilution 1:500), B_mem_ cell supernatants (1:20), or mAbs (20 µg/mL) were prepared. Serum and mAbs underwent two- and threefold eight-point serial dilutions, respectively, before being added to plates in duplicate and incubated for 1 h at 37 °C. HA-specific IgG was detected using horseradish peroxidase (HRP)-conjugated goat anti-human IgG (catalog no. 2040-05, Southern Biotech; 1:4,000) for 1 h at 37 °C. After washing, plates were developed with 0.1% ABTS solution containing 0.05% H_2_O_2_ for 20 min at 37 °C. The reaction was quenched with 1% SDS, and absorbance at 414 nm was recorded via a PowerWaveXS plate reader (BioTek). Binding activity was quantified as the area under the curve (AUC).

### 2.10 Hemagglutination inhibition assay

Functional antibody titers were measured via the HAI assay using turkey erythrocytes, following protocols adapted from the CDC and WHO (27).

To eliminate nonspecific inhibitors, sera were treated with receptor-destroying enzyme (catalog no. 370013, RDE; Denka Seiken, Co., Japan) at a 3:1 ratio, incubated overnight at 37 °C, and heat-inactivated at 56 °C. RDE-treated sera were then diluted to a 1:10 starting concentration in PBS and twofold serially diluted across v-bottom microtiter plates (catalog no. 651101, Greiner Bio-One, Monroe, NC, USA).

For mAb testing, antibodies were initially diluted to 20 µg/mL and then twofold serially diluted in PBS across v-bottom microtiter plates.

For both RDE-treated polyclonal sera and mAbs, each well received 25 μL of ether-treated IBV, standardized to approximately 8 HAU/50 μL. Following a 30-min incubation at RT, turkey erythrocytes (0.75% in PBS; Lampire Biologicals) were added. These erythrocytes were maintained at 4 °C and utilized within 72 h of preparation. The HAI titer was recorded as the reciprocal of the highest dilution (or lowest mAb concentration) exhibiting complete inhibition of hemagglutination. In accordance with WHO and European Committee for Medicinal Products standards, seroprotection was defined as a polyclonal antibody titer ≥ 1:40, while seroconversion required a fourfold increase from baseline to a minimum of 1:40. Subjects with titers < 1:40 were classified as seronegative.

### 2.11 Animal studies

BALB/c mice (female, 8–10 weeks of age), antibody negative for circulating IAVs (H1N1 and H3N2) and IBVs, were purchased from Jackson Laboratory (Bar Harbor, ME, USA), housed in microisolator units, and fed *ad libitum*. Animal procedures were performed in strict accordance with protocols approved by the Cleveland Clinic Institutional Animal Care and Use Committee (IACUC) and adhered to U.S. Department of Agriculture guidelines for the care of laboratory animals. Following a minimum 72-h acclimation period, mice were randomly assigned to three groups (*n* = 10 per group) for use in either prophylactic or therapeutic challenge studies.

IBV prophylactic and therapeutic studies: 10 mice per group were prophylactically (24 h before infection challenge) or therapeutically (24 h after infection challenge) administered intraperitoneally with 1.5 mg/kg of mAb #46 or of the isotype control mAb CR3022 (28). Intranasal infection was performed with 10^7^ plaque-forming units (PFU) of the IBV WA/19 strain. Infection was performed following mouse anesthesia by administering 2%–5% isoflurane (Piramal Critical Care, Bethlehem, PA, USA) by inhalation.

Body weight, clinical signs, and survival rates were recorded for 14 days post-infection (p.i.), with observers blinded to the treatment groups. Three randomly selected mice per group were sacrificed 3 days p.i. for determining lung viral titers. For the clinical signs, the following scoring system was used: score = 3, weight loss > 25% or severe respiratory distress; score = 2, dyspnea or failure to respond to external stimuli; score = 1, weight loss of 15%–25% or lethargy. Mice with a clinical score >3 were humanely euthanized. Euthanasia was performed in accordance with the American Veterinary Medical Association (AVMA) guidelines for the euthanasia of animals (2020 edition), with a 30%–70% CO_2_ flow rate of chamber volume per minute followed by cervical dislocation.

### 2.12 Determination of lung viral titer

For lung virus titering, a plaque assay was performed similarly to previously described protocols (29). Two days prior to the assay, low-passage MDCK cells were seeded into six-well plates (catalog no. 657160, Greiner Bio-One) at a density of 3.8 × 10^5^ cells per well. Diluted samples were overlaid in 100 μL of supplemented DMEM and incubated for 1 h at RT. After washing the monolayers, an overlay of Modified Eagle Medium containing 0.8% agarose (Lonza) was applied, and the plates were incubated for 72 h at 37 °C, 5% CO_2_. Following incubation, the agarose was removed, and cells were fixed with 10% buffered formalin and stained with 1% Crystal Violet (catalog no. S25275A, Fisher Science Education, Pittsburgh, PA, USA) for 15 min. After rinsing with distilled water and drying the plates, plaques were quantified to calculate the PFU per gram of lung tissue.

### 2.13 Neutralization assay

The focus reduction assay (FRA) was performed similarly to previously described protocols (20). MDCK-SIAT1 cells were seeded in 96-well plates (catalog no. 655101, Greiner Bio-One) at a density of 2.5–3 × 10^5^ cells/mL 1 day prior to the assay. On the following day, cell monolayers were rinsed with PBS (Corning) and incubated with 50 μL of twofold serially diluted mAbs. The dilutions originated from a 20-μg/mL stock prepared in virus growth medium supplemented with 1 μg/mL of L-(tosylamido-2-phenyl) ethyl chloromethyl ketone TPCK-treated trypsin (catalog no. 20233, Thermo Fisher Scientific).

Afterwards, 50 μL of MA/12 virus standardized to 1.2 × 10^4^ focus-forming units (FFU) per milliliter and corresponding to 600 FFU per 50 μL was added to each well, including control wells. Following a 2-h incubation period at 37 °C with 5% CO_2_, the cells in each well were then overlaid with 100 μL of equal volumes of 1.2% Avicel RC/CL (Type RC581 NF; FMC Health and Nutrition, Philadelphia, PA, USA) in 2×MEM (catalog no. 11430030, Thermo Fisher Scientific) containing 1 μg/mL TPCK-treated trypsin, 0.1% BSA, and antibiotics. Plates were incubated for 18–22 h at 37 °C in 5% CO_2_. Following incubation, overlays were removed, and monolayers were rinsed with PBS to eliminate residual Avicel. Cells were fixed with ice-cold 4% formalin for 30 min at 4 °C, then permeabilized with 0.5% Triton X-100 (catalog no. X100, Sigma-Aldrich, St Louis, MO, USA) in PBS/glycine for 20 min at RT. After three washes with PBS supplemented with 0.1% Tween 20 (PBST), the plates were incubated for 1 h with an anti-IBV nucleoprotein mAb (IRR, FR-52) prepared in ELISA buffer [PBS with 10% horse serum and 0.1% Tween 80 (catalog no. 28329, Thermo Fisher Scientific)]. Following another wash cycle, a goat anti-mouse peroxidase-labeled IgG secondary antibody (catalog no. 5220-0341, SeraCare, Milford, MA, USA) was applied for 1 h at RT. Infectious foci were visualized using TrueBlue substrate (catalog no. 5510-0030, SeraCare) containing 0.03% H_2_O_2_ for 10–15 min, and the reaction was quenched with five distilled water washes. Upon drying, foci were enumerated via an S6 macro ELISpot reader (ImmunoCapture 6.4.87 software), and neutralization potency was determined across a mAb dilution range of 10 to 0.02 μg/mL.

### 2.14 Kinetic assay by biolayer interferometry

The binding kinetics of mAbs for the HA protein was determined by biolayer interferometry (BLI) using an Octet RH16 system and Streptavidin (SA) biosensors (Sartorius GmbH, Göttingen, Germany). The affinity (equilibrium dissociation constant, *K*_D_) values of mAbs were determined against the Brisb/08, PH/13, and AU/21 rHA used at 25 μg/mL. Briefly, biosensors were hydrated for 10 min in kinetic buffer (1× PBS supplemented with 0.5% BSA), which also served as the diluent for all antibodies and antigens, as well as the medium for baseline and dissociation steps. MAb affinity was measured using an automated five-step program at 25 °C with 1,000 rpm agitation. The sequence consisted of an initial 60-s reference baseline, a 300-s loading phase, and a secondary 60-s baseline, followed by a 300-s association phase (*k*_on_) across four mAb concentrations (333.3, 166.7, 83.3, and 41.7 nM). Dissociation (*k*_off_) was subsequently monitored for 1,500 s. Data were processed using Data Analysis Studio software (version 12.2), and the *K*_D_ was calculated as the ratio of *k*_off_/*k*_on_ using a 1:2 bivalent analyte model.

### 2.15 Epitope binning by biolayer interferometry

The panel of mAbs was reciprocally competed for binding using the IBV WA/19 rHA protein on the BLI Octet RH16 system (Sartorius) as previously described (30). Epitope competition assays were performed using Anti-penta-HIS (HIS1K) biosensors (Sartorius) pre-hydrated in kinetics buffer (PBS + 0.5% BSA + 0.05% Tween 20). After an initial 60-s baseline, biosensors were loaded with 50 μg/mL of recombinant protein for 300 s. For the association phase, sensors were immersed in a competing mAb (100 μg/mL) for 300 s, followed by immersion in a second “probe” mAb (100 μg/mL) for an additional 300 s. Between competition sets, biosensors were regenerated via three alternating cycles of 0.1 M glycine (pH 2.5) and kinetics buffer. The degree of competition was quantified as percent inhibition using the following formula: percent inhibition = 100 × [(signal of probe mAb alone − signal of probe mAb with competitor mAb)/signal of probe mAb alone]. Competition levels were categorized as complete (≥73%), moderate (<73% or ≥45%), or none (<45%).

### 2.16 Antibody-dependent cell-mediated cytotoxicity assay

An antibody-dependent cell-mediated cytotoxicity (ADCC) assay was performed using the ADCC reporter bioassay kit (catalog no. 7018, Promega, Madison, WI) according to the manufacturer’s instructions and as previously described (20, 26). In brief, Immulon 4HBX plates were coated overnight at 4°C with 50 μL per well of a solution of PBS containing 5 μg/mL Brisb/08 rHA. MAbs, including an irrelevant IgG1 isotype control mAb (31), were fivefold serially diluted starting from a final concentration of 20 μg/mL in assay buffer, consisting of RPMI 1640 medium (provided in the kit) supplemented with 4% heat-inactivated super low IgG FBS (provided in the kit). MAb dilutions were added to the plates along with 5 × 10^4^ ADCC bioassay effector cells per well, achieving a final volume of 75 µL. Following a 6-h incubation at 37 °C 5% CO_2_, the plate contents were transferred to 96-well white polystyrene plates (Corning). To each well, 75 µL of Bio-Glo luciferase reagent was added and allowed to react for 5 min at RT. Luminescence was recorded using a GloMax-96 microplate luminometer (Promega), with ADCC activity reported as relative light units (RLU). The AUC of relative luminescence units obtained at different mAb dilutions was then calculated using GraphPad Prism v10.6.0 software (GraphPad Software, San Diego, CA).

### 2.17 Peptide scanning

Immulon 384-well plates (Thermo Fisher Scientific) were coated with 5 μg/well of overlapping 13- or 15-mer peptides spanning the Brisb/08 HA ectodomain (BEI Resources, catalog no. NR-19247) in PBS for 16 h at 4 °C in humidified chambers. Plates were blocked with blocking buffer (2% BSA, 1% gelatin in PBS/0.05% Tween 20) for 2 h at 37 °C. MAb #46 was diluted to 10 µg/mL concentration in blocking buffer and added to the assay plate in duplicate and incubated for 1 h at 37 °C in humidified chambers. Plates were washed four times with PBS, and HA-specific IgG was detected using HRP-conjugated goat anti-human IgG (catalog no. 2040-05, Southern Biotech) at a 1:4,000 dilution and incubated for 1 h at 37 °C. Plates were then washed three times with PBS prior to development with 100 μL of 0.1% ABTS solution with 0.05% H_2_O_2_ for 20 min at 37 °C. The reaction was quenched by the addition of 50 μL 1% (w/v) SDS. Absorbance at 414 nm was measured using a PowerWaveXS plate reader (BioTek).

### 2.18 Expression and purification of COBRA BC2 for single-particle cryo-EM

A gene containing the ectodomain of COBRA BC2 (11) appended by a thrombin cleavage site, T4 fibritin “Foldon” trimerization domain, hexahistidine tag, and StrepTag II was generated through a combination of gene synthesis and Gibson assembly (32). The construct was cloned into a derivative of the pcDNA3.1 vector (33) and maxi-prepped free of endotoxin for transfection in mammalian cells (Invitrogen). Transient transfection of CHO-S was performed by electroporation (Maxcyte STX, Rockville MD, USA) following the manufacturer’s protocol, and the expression continued for 10 days using previously described culture conditions (33, 34). The cultures were harvested by centrifugation; the supernatant was passed through a 0.2-μm filter and then loaded onto a HisTrap excel column (Cytiva) pre-equilibrated with Buffer A (500 mM NaCl and 20 mM Tris, pH 8.0) using an ÄKTA Pure Chromatography system (Cytiva). The column was washed with Buffer A followed by a gradient elution to Buffer B (500 mM NaCl, 20 mM Tris, pH 8.0, and 250 mM imidazole). Fractions containing COBRA BC2 as assessed by SDS-PAGE were pooled, concentrated, and dialyzed into Tris buffered saline (TBS; 150 mM NaCl and 50 mM tris, pH 8). A subset of the dialyzed sample was subjected to an overnight thrombin digestion and further purified by size exclusion chromatography on a Superdex200 10/300 column (Cytiva) to separate the HA from the Foldon/tags. Fractions containing HA based on SDS-PAGE analysis were pooled, concentrated, and kept at 4°C until further use.

### 2.19 Expression and purification of mAb #46 for single-particle cryo-EM

Plasmids containing the HC and LC genes of mAb #46 were maxi-prepped and endotoxin-free DNA (Invitrogen) was transiently transfected by electroporation (Maxcyte STX) into CHO-S cells with a 1:1 ratio by weight of the HC: LC plasmids of each antibody. After 8–10 days, the cultures were harvested and the clarified, filtered media was purified by affinity chromatography on a Protein G column (Cytiva). To accomplish this, the supernatant was diluted 1:1 in PBS (137 mM NaCl, 10 mM Na_2_HPO_4,_ 2.7 mM KCl, and 1.8 mM KH_2_PO_4_, pH 7.4) and applied to a column pre-equilibrated in PBS. The column was then washed with 15 column washes of wash buffer (PBS). Bound IgG was eluted with 0.1 M glycine-HCl (pH 2.7), collecting fractions directly into neutralizing buffer (1 M Tris-HCl, pH 9.0). Fractions containing mAb were pooled, dialyzed in PBS, then snap-frozen in liquid nitrogen for storage at −80 °C.

The Fab fragment of mAb #46 was generated by digestion with papain (Pierce Fab Preparation Kit, Thermo Fisher Scientific). The recovered Fabs were polished by size exclusion chromatography on a Superdex 200 10/300 column (Cytiva). Both Fabs were then added to COBRA BC2 in a 1.1:1 Fab:HA molar ratio and incubated at 4°C overnight to allow for complex formation. The sample was subsequently concentrated and applied to a Superose 6 10/300 column to separate the complex from free Fab. Fractions containing both HA and Fab were assessed by SDS-PAGE, pooled, and concentrated to 1.566 mg/mL, then stored at 4°C.

### 2.20 Single-particle cryo-electron microscopy

The sample was adjusted to a concentration of 0.4 mg/mL and deposited onto a glow-discharged Quantifoil R1.2/1.3–200 mesh grid. Blotting and vitrification in liquid ethane were performed with a VitroBot Mark IV (Thermo Fisher Scientific) at 4°C at 100% humidity. Screening was conducted at the UCSC Biomolecular cryo-EM facility using a Thermo Fisher Scientific (TFS) Glacios cryo-TEM operated at an accelerating voltage of 200 kV and coupled to a Gatan K2 Summit direct electron detector. Data collection was performed at the Pacific Northwest Center for Cryo-EM (PNCC #160263) on a TFS Krios G3i operating at 300 kV coupled to a Falcon 4i detector and SelectrisX energy filter. The data were collected in EPU in counting mode at a nominal magnification of 105,000×, a pixel size of 0.5727 Å/pixel, an exposure time of 2.32 s, and a total dose of 50 electrons/Å^2^. Both untilted (2,171 movies) and 30° tilted (32,854 movies) datasets were collected to mitigate the impact of preferential orientation. The movies were combined during preprocessing in CryoSPARC v4.5.3 for Patch Motion Correction and Patch CTF estimation (35, 36). Following exposure curation, the remaining 21,142 micrographs were denoised, and Blob picking was performed on a subset of 5,000 micrographs. An initial 1,435,483 particles were extracted with 2× binning by Fourier cropping and applied to multiple rounds of 2D classification. An *ab initio* reconstruction with five classes was performed with 576,067 particles, followed by heterogeneous refinement with all five volumes with the particles from the top three classes. The particles corresponding to the best three volumes in the heterogeneous refinement were used to perform an additional *ab initio* reconstruction with a single class followed by homogeneous refinement. This volume was used to generate 2D templates to perform template picking across all the curated micrographs.

Additional templates were generated from a 20-Å filtered volume derived from EMD-8731 to improve the representation of side views. Following particle picking and curation, 7,187,918 particles were extracted with 8× binning by Fourier cropping. Particle sorting was performed in 2D and 3D by multiple rounds of 2D classification and *ab initio* reconstruction with multiple classes. The top 1,184,747 particles were re-extracted and subjected to multiple rounds of 2D classification. A set of 1,103,398 particles from the final selected 2D classes were put into an *ab initio* reconstruction with 4 classes followed by heterogeneous refinement. The volume and particles associated with Class 1 were advanced to homogeneous refinement in C1 symmetry. Following re-extraction of the aligned particles with a tighter box size, a series of homogeneous and non-uniform (NU) refinements (37) were performed, followed by another round of 3D classification. The five resulting classes were regrouped into two superclasses, one containing four Fabs (three of #46, one of #3978) and the other with three Fabs (two of #46, one of #3978) (38). Superclass 0 (four Fabs) with its 209,227 associated particles was further refined by a series of NU refinements with C1 and C3 symmetry, followed by rounds of Global and Local CTF refinement interspersed by NU refinement. Masks were subsequently generated to perform Local refinement on the head domain of HA with the #46 Fab and particle subtraction to remove the flexible Fab constant domains and the #3978 Fab. Following additional polishing by Local refinement and Local CTF refinement, the half maps were subjected to anisotropy correction with spIsoNet (39). The corrected half maps were subsequently used to generate a sharpened map for interpretation and model building.

Initial model building was performed with ModelAngelo in sequence mode (40). The built model was used to assist in the placement of a single BC2:#46 heterodimer derived from PDB 9NJ3 into the map at 2.5 Å overall resolution. Model building and refinement were performed iteratively in Coot, ISOLDE in ChimeraX, and Phenix (41–43). The model was expanded to C3 symmetry prior to the final refinement. Model validation was performed using MolProbity (44) for protein geometry and Privateer in the CCPEM 1.7.0-rc interface (45–48) for glycan structure. The maps and model associated with the structure were deposited in the Electron Microscopy Databank and Protein Data Bank, respectively, with accession numbers EMD-49477 and PDB 9NJA. Parameters related to data collection and model refinement are reported in **Table 3**. Structural images were generated using UCSF ChimeraX (49).

**Table 3:** Single-particle cryo-EM data collection, refinement, and validation statistics.

COBRA BC2:#4 (EMD-49477) (PDB 9NJA)	
Data collection and processing	
Microscope	Krios G3i
Voltage (kV)	300
Energy filter Slit width (μm)	SelectrisX 10 eV
Camera	Falcon 4i
Magnification	105,000
Pixel size (Å)	0.5727
Electron exposure (e^–^/Å^2^)	50
Defocus range (μm)	0.5-2.8
Initial particle images (*n*)	1,103,398 (selected 2D classes)
Final particle images (*n*)	209,227 (final 3D refinement)
Symmetry imposed	C3
Overall map resolution (Å) FSC threshold	2.50 0.143
Min/max resolution (Å)	5.83/2.06
Map sharpening *B* factor (Å^2^)	−93.08
Refinement	
Initial model used	PDB 9NJ3
Map-model FSC resolution (Å) FSC threshold	2.9 0.5
Model composition	
Non-hydrogen atoms Protein residues Glycan residues	11,688 1,518 12
Model *B* factors (Å^2^)	
Protein Glycan	68.71 112.49
R.m.s. deviations	
Bond lengths (Å) Bond angles (°)	0.002 0.476
Validation	
MolProbity score Clashscore Poor rotamers (%)	1.16 3.76 0.96
CaBLAM outliers (%)	0.82
*Q*-score (overall)	0.565
Ramachandran plot	
Favored (%) Allowed (%) Disallowed (%)	98.39 1.61 0.00

### 2.21 Statistical analysis

For the assessment of the participants’ polyclonal serum binding and HAI activity and D7 PB-derived ASCs, differences across age groups were evaluated using a two-way analysis of variance (ANOVA; mixed model) and a Tukey test for the correction of the multiple comparisons.

For the mouse studies, significance in body weight, clinical score, and lung viral titer differences was evaluated using an ordinary one-way ANOVA.

For the ADCC assay using the mAbs, significance of the differences in their levels was evaluated through multiple comparisons and using an ordinary one-way ANOVA, where the ADCC activity mean value of each mAb was compared to the mean value of every other mAb.

All statistical analyses were performed using GraphPad Prism V.10.6.0 software, and a *p*-value <0.05 was considered statistically significant.

## 3 Results

### 3.1 Polyclonal antibody profiling of participants vaccinated with the split-inactivated influenza vaccine

**Figure 2:**
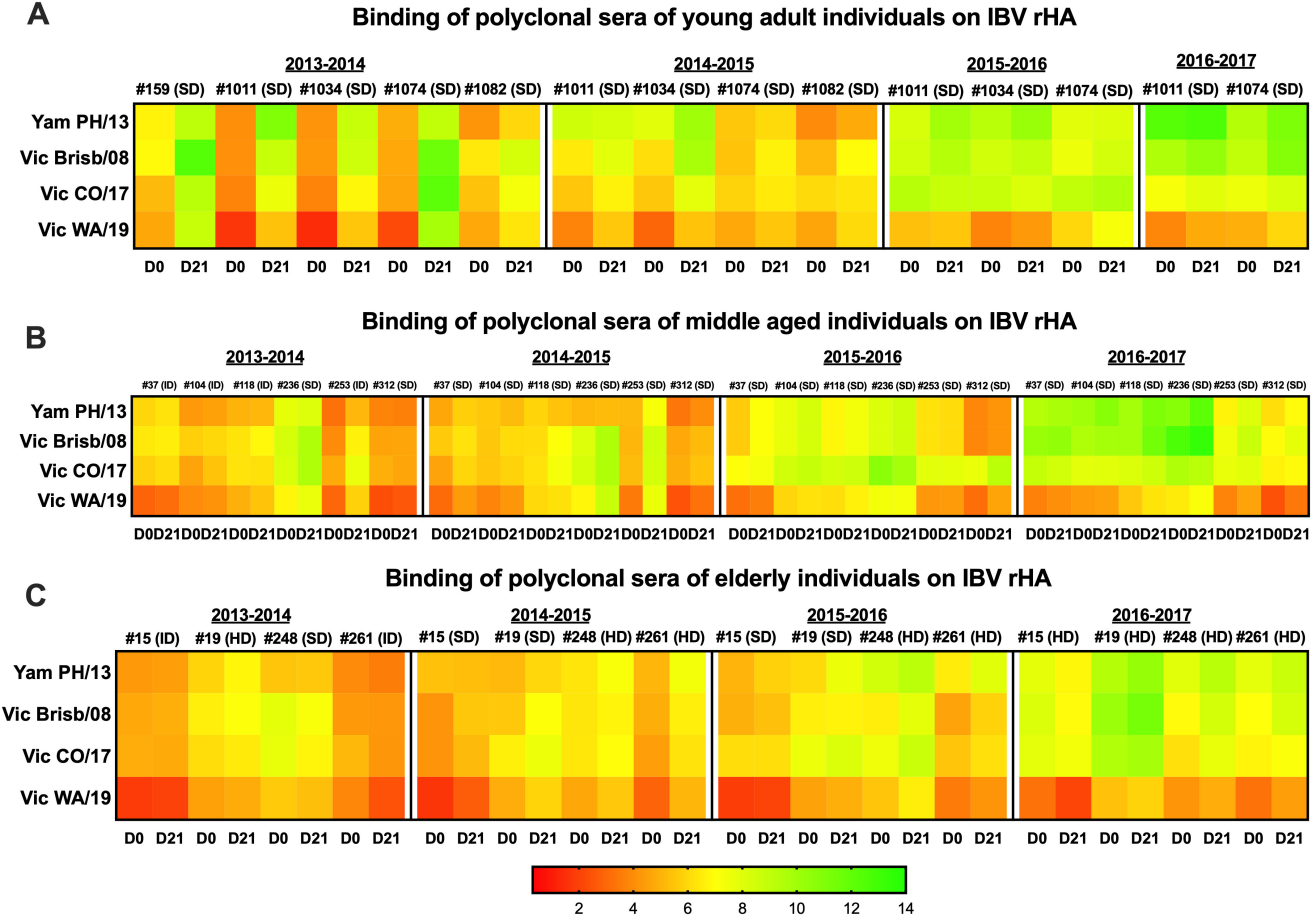
Heatmaps of binding of polyclonal sera collected from study participants belonging to different age groups. Young adults **(A)**, middle aged **(B)**, and elderly **(C)**, at baseline (D0) and 21 days (D21) following administration of QIV: standard dose (SD); intradermal (ID) or high dose (HD). Breadth of binding was determined by ELISA against a panel of rHA representing historical and recent IBV vaccine strains belonging to the Yamagata (Yam) and Victoria (Vic) lineages. Binding is expressed as the heatmap of the AUC of the OD values obtained from the serum dilutions of each participant ID against the corresponding IBV HA. ELISAs were performed in duplicate for two independent experiments. Results represent one experiment performed in duplicate. AUC values are represented by colors as depicted in the color legend.

Polyclonal sera tested against a panel of rHA belonging to the Yamagata lineage PH/13 and Victoria lineage Brisb/08, CO/17, and WA/19 revealed an overall higher magnitude of binding in young individuals compared to the middle-aged and elderly, with antibody responses mostly pronounced at D21 compared to D0 (**Figure 2**; **Supplementary Figure 1**, and **Supplementary Table 1**). However, this difference reached statistical significance especially at D21 in the 2013–2014 and 2015–2016 (for PH/13 compared to the middle-aged group) seasons only (*p* > 0.05). A longitudinal increase of the magnitude of response against the same panel was observed in all the age populations and especially in donors #1011 and #1074 (young); #37, #104, #118, and #236 (middle age); and #19 (elderly), especially in the last analyzed season (2016–2017).

**Figure 3:**
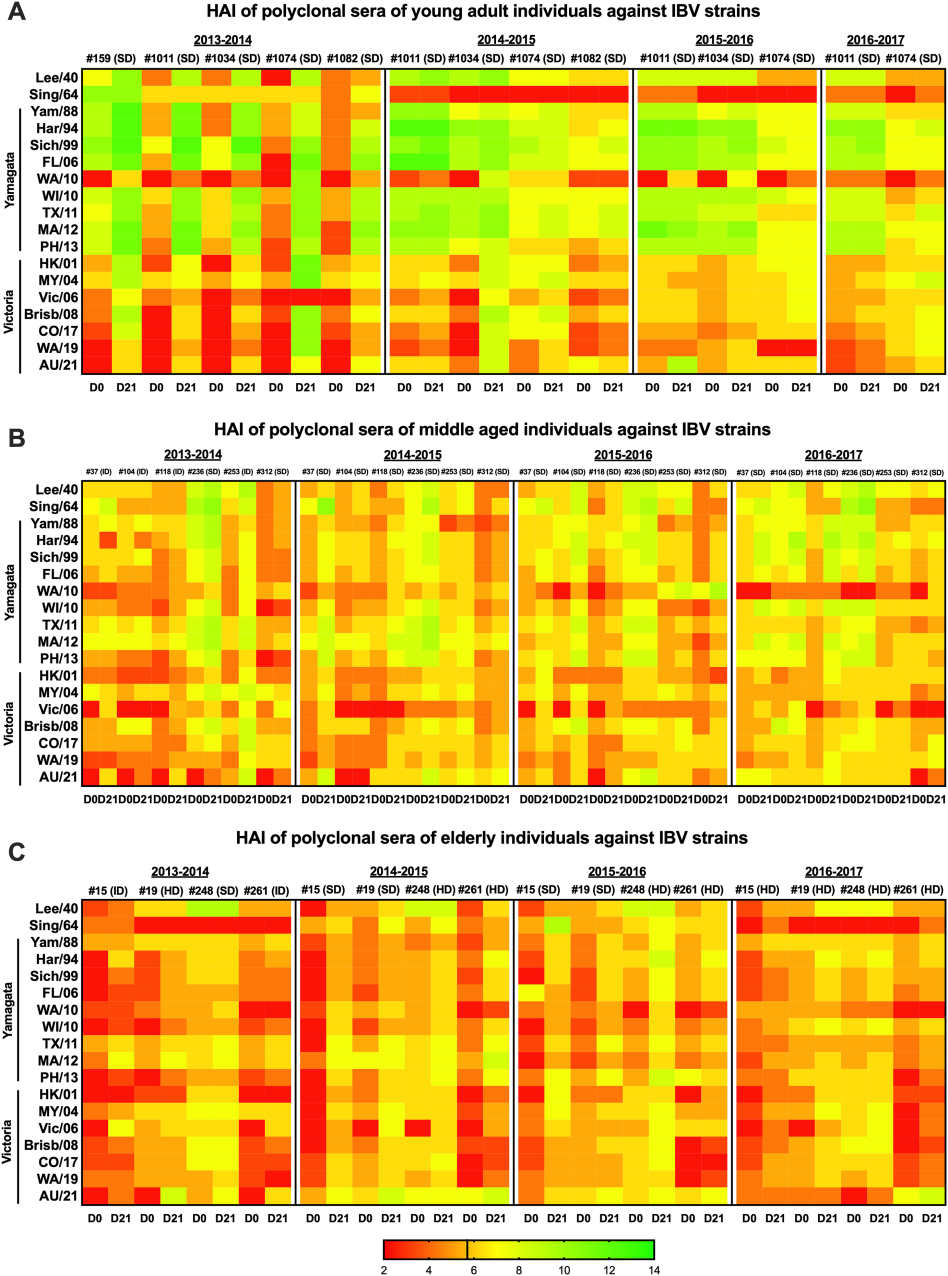
Heatmaps of HAI activity of polyclonal sera collected from study participants belonging to different age groups. Young adults **(A)**, middle aged **(B)**, and elderly **(C)**, at baseline (D0) and 21 days (D21) following administration of QIV: standard dose (SD); intradermal (ID) or high dose (HD). Breadth of functional activity was determined by HAI assay against a panel of IBV isolates representing historical and recent vaccine strains. HAI is expressed as the heatmap of the Log2 of the reciprocal dilution of the last serum dilution point that inhibited the hemagglutination. The vertical line in the HAI color legend indicating the 5.32 Log2 value corresponds to the 1:40 seroprotective threshold value. HAIs were performed in duplicate for two independent experiments. Results represent one experiment performed in duplicate.

Similar to the binding activity, polyclonal sera tested against a panel of vaccine strains belonging to the Yamagata and Victoria lineages revealed an overall higher magnitude of HAI activity at baseline (D0) and at D21 in the young individuals especially against the Yamagata strains compared to the middle-aged and elderly, again with antibody responses more pronounced at D21 compared to D0, especially against strains encompassing the 1964–2013 (2013–2014, 2015–2016, and 2016–2017 seasons) and 1940–2010 (2014–2015 season) eras (*p* < 0.05) (**Figure 3**; **Supplementary Figure 2**, **Supplementary Table 2**).

An overall lower response without a statistically significant difference across age groups was instead observed for the Victoria lineage strains except for the 2008–2019 strains (at D0_2013–2014_) and the HK/01 (D21_2013–2014_ and D0_2015–2016_), MY/04 (D0_2014–2015_), Vic/06 (D0_2015–2016_), and Brisb/08 at D21 (2013–2014 and 2014–2015 seasons) for young individuals.

### 3.2 Analysis of the PB response following QIV

To understand the breadth of the polyclonal antibody response at the peak of the PB expansion, the frequency of IgM, IgG, and IgA ASCs from D7 PBMC samples longitudinally collected following QIV vaccination was evaluated. While no statistical difference was observed across age groups in the different seasons in terms of total ASC for IgG, IgM, and IgA (**Supplementary Figure 4A**), a higher frequency of HA PH/13+ and BC2+ IgG and IgA ASCs was observed across all the tested seasons and across all age groups, especially from samples collected in the 2016–2017 season (**Supplementary Figures 3A, B**). In particular, the IgG ASC frequency was statistically significantly higher in young individuals compared to middle-aged individuals (against PH/13 in 2014–2015) and for both IgG and IgM in 2015–2016 (also against BC2) (*p* < 0.05).

### 3.3 Analysis of the antibody profile of the B_mem_ cell response following QIV

To understand how vaccination influences the B_mem_ compartment, PBMCs from longitudinally collected blood samples at D0 and D21 were subjected to *in vitro* stimulation, and the resulting antibody reactivity from culture supernatants against IBV historical and recent IBV vaccine sequence rHAs as well as against COBRA IBV rHAs was assessed by ELISA (**Supplementary Figure 3B**, **5**).

Across all age groups, pre-existing B_mem_-derived antibody levels were statistically significantly higher at D21 in young individuals against the IBV vaccine strains HK/01, Brisb/08, and BC/17 at D0 (2013–2014) and against PH/13 (2014–2015) and for middle-aged compared to elderly individuals in 2015–2016 (**Supplementary Table 3**). A generally lower magnitude of binding was observed against more recent vaccine strains and COBRA HAs (**Supplementary Figures 3**, **5**).

### 3.4 Binding, HAI, and neutralizing activity of IBV-specific mAbs

Following the IgG1 PB antibody repertoire sequencing using the PBMC samples collected at D7 from these individuals, a high-throughput expression and screening of the corresponding mAbs revealed the presence of IBV HA-specific antibodies from participant #253 (25, 50, 51).

**Figure 4:**
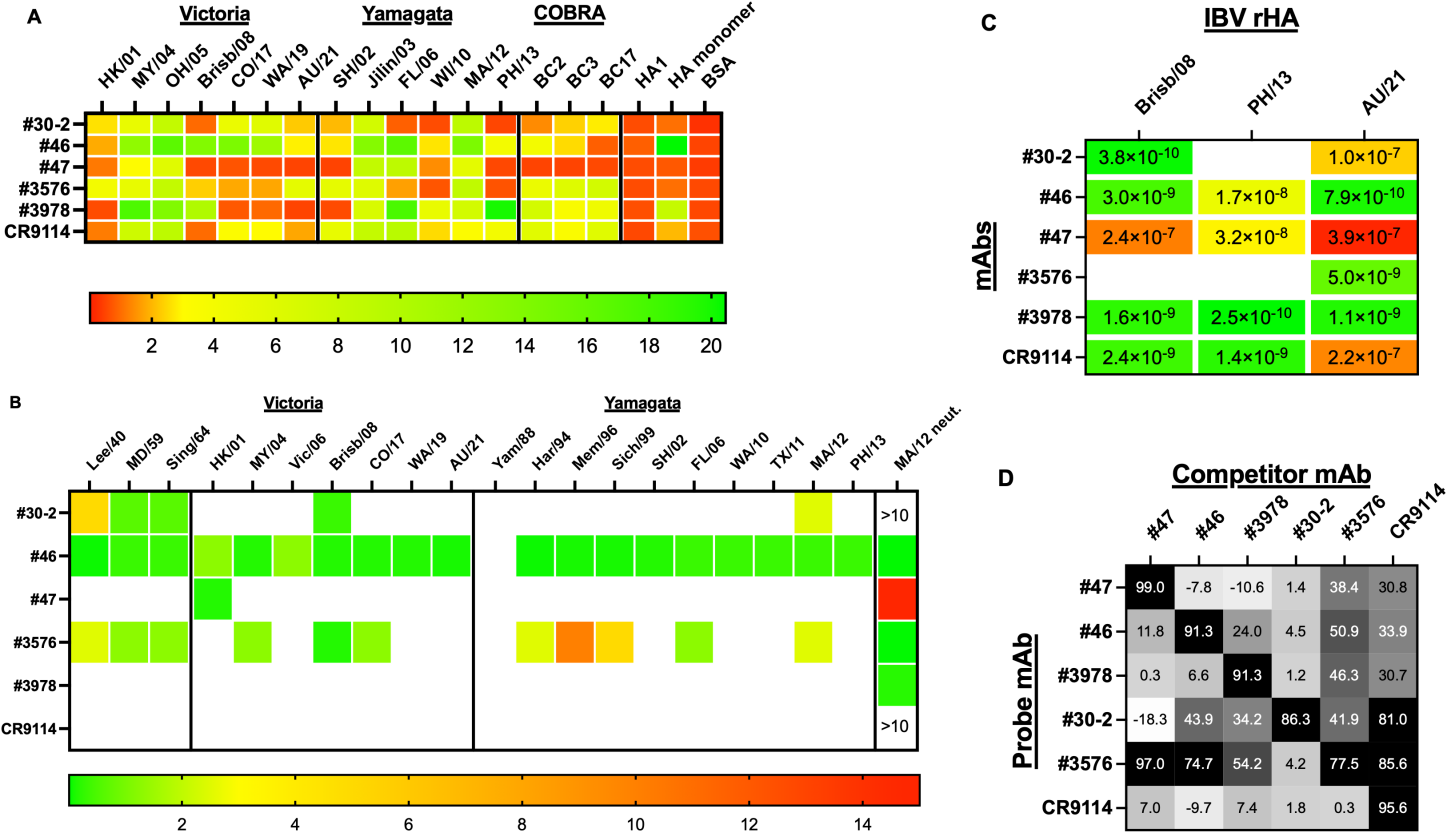
Heatmaps of binding and functional activity of IBV HA-specific mAbs. **(A)** Breadth of binding was determined by ELISA against a panel of recombinant HA representing historical and recent IBV vaccine strains belonging to the Victoria and Yamagata lineages as well as against the HA monomers and the HA1 region from MA/12. BSA was used as a negative control. The binding activity of mAbs is expressed as AUC obtained from the corresponding binding curves using different mAb dilutions. **(B)** Breadth of functional activity was determined by HAI assay against a panel of IBVs representing historical and recent vaccine strains belonging to the Victoria and Yamagata lineages. HAI is expressed as the minimal tested concentration (endpoint concentration) that inhibited hemagglutination. ELISA and HAI were performed in duplicate for two independent experiments. Neutralizing activity was assessed against the MA/12 strain and IC_50_ is reported in the heatmap. Results represent one experiment performed in duplicate. **(C)** Equilibrium dissociation constants (*K*_D_), expressed as a molar (M) concentration, between each antibody and the corresponding antigens are reported as heatmap. Results represent two independent experiments performed in duplicate. **(D)** Synopsis of competition for binding of human mAb pairs. Competitive binding assays were performed using biolayer interferometry (BLI) and pairs of IBV HA-specific human mAbs. Competition values are expressed as a percentage of the inhibition of binding of the probe mAb in the presence of the competitor mAb. For the competition assays, the rHA AU/21 was used as target antigen. Results represent two independent experiments performed at four different mAb dilutions.

To confirm their specificity and determine the extent of binding breadth, mAbs were assessed for binding against a panel of Yamagata and Victoria lineage IBV rHAs, representing historical and more recent vaccine strains as well as COBRA rHAs. All the mAbs showed a breadth of binding against both Yamagata and Victoria lineage IBV rHAs (**Figure 4A**). In particular, mAb #30–2 was able to bind all the strains of the Victoria panel, with a lower extent to the Brisb/08 rHA, and of the Yamagata panel, with a lower extent to the FL/06, WI/10, and PH/13 rHAs, as well as all the IBV COBRA rHAs. No binding was observed against the HA1 region and the monomer HA.

Mab #46 showed a breadth of binding against all the Yamagata and Victoria rHA of the panel, including two of the COBRA rHAs (BC2 and BC3). Binding was also observed against the HA monomer but not the HA1 region.

MAb #3576 showed a broad binding profile with a lower potency of binding against more recent Victoria strains (CO/17 and WA/19) and some Yamagata strains (FL/06, WI/10, and PH/13). Binding of this mAb against all the tested IBV COBRA rHA was observed.

MAb #47 showed a narrower binding profile with a more pronounced binding against 2004–2005 Victoria strains and 2003–2012 Yamagata strains, and no binding against all the tested COBRA rHA.

The previously described mAbs #3978 and CR9114 showed a comparable binding profile to previously described results (38, 52). In particular, mAb #3978 showed a prominent binding profile against 2004–2008 Victoria strains and 2003–2013 Yamagata strains as well as binding against all the tested IBV COBRA rHA.

All the mAbs, except for #3978 and CR9114, were endowed with HAI activity with different breadths (**Figure 4B**). MAb #46 showed HAI activity against all the tested strains from the Victoria and Yamagata lineages except for the Yam/88 strain and a minimal inhibition concentration (MIC) ranging from 0.08 to 1.25 μg/mL and a potent neutralizing activity against the MA/12 strain (IC_50_ of 0.02 μg/mL). MAb #3576 showed HAI activity against MD/59 and 2004–2017 Victoria strains and against 1994–1999, FL/06, and MA/12 Yamagata strains. Neutralization against the MA/12 strain was also confirmed (IC_50_ of 0.02 μg/mL).

MAbs #30–2 and #47 showed the narrowest HAI profile with #30–2 having HAI activity against the MD/59 and Brisb/08 Victoria strain (MIC 0.6–0.3 μg/mL) and the MA/12 Yamagata strain (MIC 2.5 μg/mL) and #47 only against the HK/01 Victoria strain (MIC 0.16 μg/mL).

### 3.5 Affinity and epitope binning of mAbs

Binding of IBV HA-reactive mAbs to Victoria Brisb/08 and AU/21 and Yamagata PH/13 HA antigens was confirmed, and their affinity was assessed by BLI. As shown in **Figure 4C**, four out of six IBV HA-reactive mAbs showed dissociation constant (*K*_D_) values in the nanomolar range (0.385–2.978 nM for mAbs #30-2, #46, #3978, and CR9114) against the Brisb/08 rHA, while mAb #47 showed a higher *K*_D_ value close to the micromolar range (235.6 nM). For mAb #3576, a *K*_D_ was not determined due to the low binding activity of this mAb against this rHA. Four out of six mAbs showed *K*_D_ values in the nanomolar range (0.254–32 nM for mAbs #46, #47 #3978, and CR9114) against the PH/13 rHA, while for mAbs #30–2 and #3576, a *K*_D_ was not determined due to the low binding activity of these mAbs against this rHA. The *K*_D_ values for three out of six mAbs were in the nanomolar range (0.788–5.031 nM for mAbs #46, #3576, and #3978) against the AU/21 rHA, while for mAbs #30-2, #47, and CR9114, a higher *K*_D_ value close to the micromolar range (100–385.2 nM) was observed.

To map the regions bound by IBV HA-specific mAbs, reciprocal competition assays were performed to assess possible overlap in their epitope recognition. As shown in **Figure 4D**, an asymmetrical competition was observed when mAbs #46 and #47 were used as competitors against mAb #3576, with mAbs #46 and #47 showing a complete competition compared to a moderate competition observed when mAb #3576 was used as a competitor. Another asymmetrical competition was observed between mAb CR9114 when used as a competitor against mAbs #30–2 and #3576 with a complete competition shown by mAb CR9114 when used as a competitor, and no competition was observed when mAbs #30–2 and #3576 were used as competitors. A moderate symmetrical competition was instead observed between mAbs #3576 and #3978.

### 3.6 ADCC of mAbs

**Figure 5:**
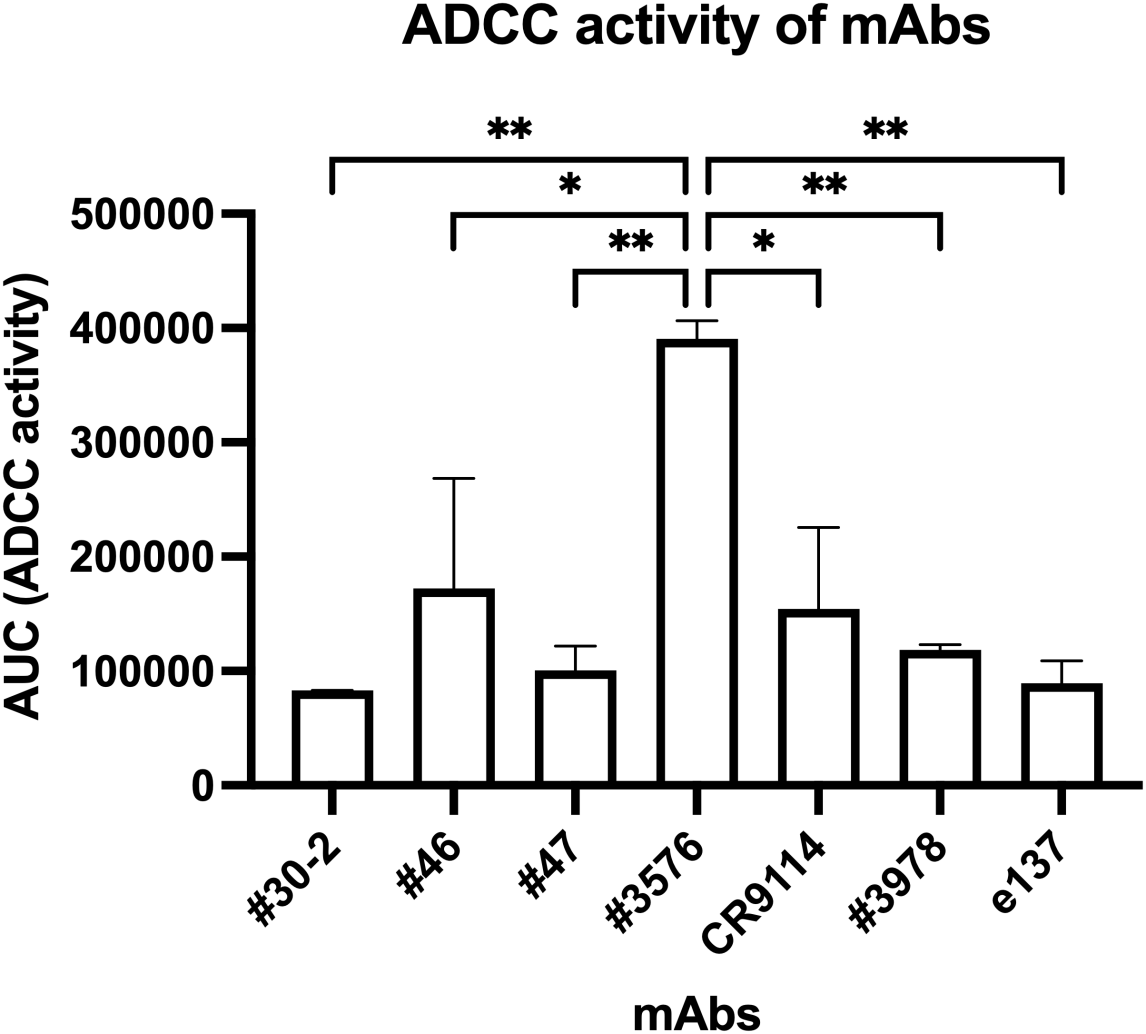
ADCC of IBV HA-specific human mAbs. ADCC activity of mAbs is reported as AUC of luciferase activity generated by ADCC reporter cells following incubation with different mAb dilutions against the Brisb/08 IBV HA. As a negative control, an irrelevant IgG1 isotype mAb (e137) was used. *p < 0.05; **p < 0.01. Results represent one experiment performed in duplicate.

IBV HA-specific mAbs were also assessed for ADCC activity to explore if they were endowed with Fc-mediated functions. As shown in **Figure 5**, none of the IBV HA-specific mAbs exhibited ADCC activity compared to the negative control mAb (e137), except for mAb #3576, which showed a significantly higher (*p* < 0.05) ADCC activity compared to all the other mAbs, including a fourfold difference with e137.

### 3.7 Single-particle cryo-EM structure of antibody #46 with COBRA BC2

**Figure 6:**
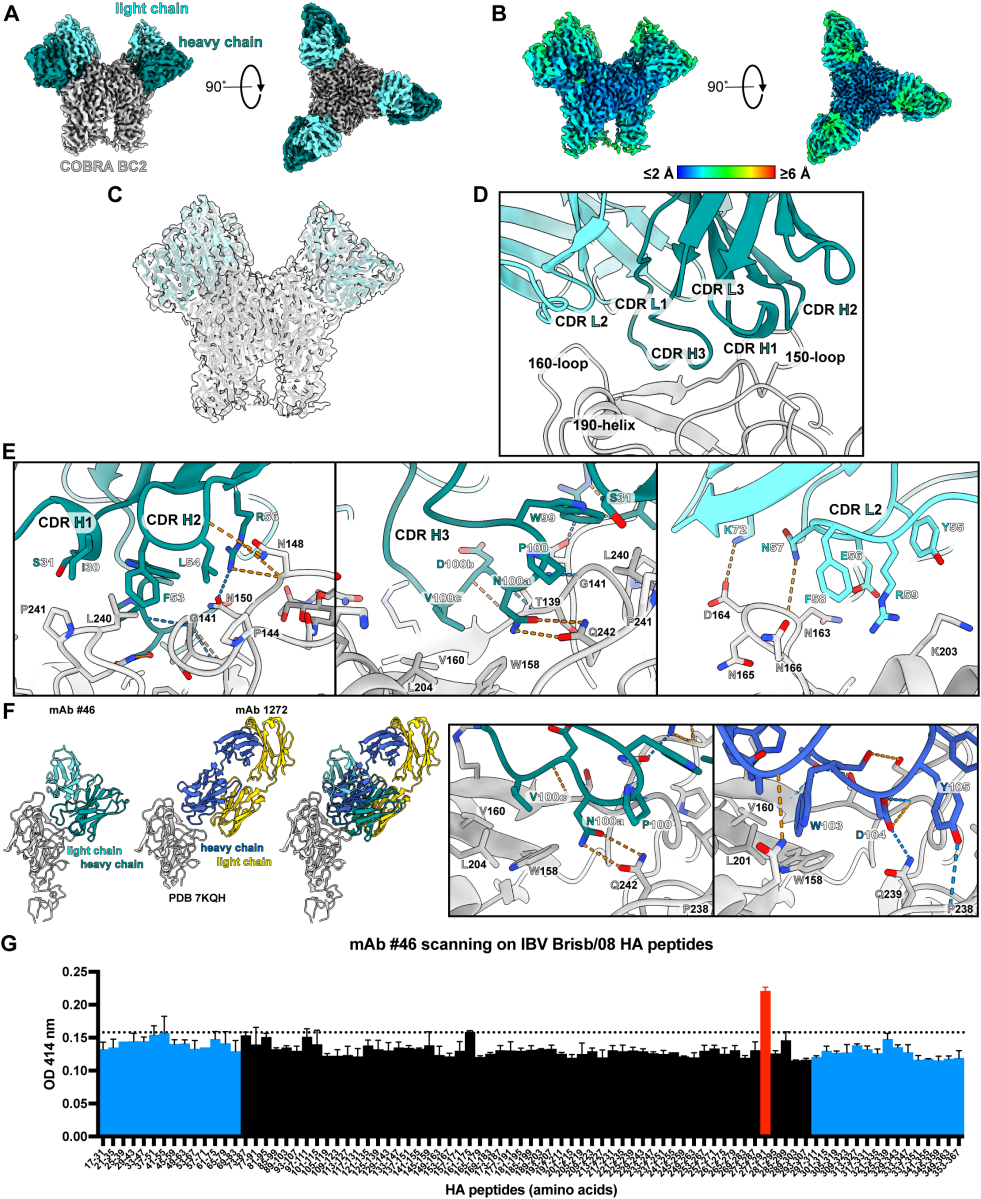
Structure of mAb #46 bound to COBRA BC2. **(A)** Single-particle cryo-EM map of COBRA BC2 bound by the Fab fragment of mAb #46. The regions corresponding to the HC and LC are colored teal and cyan, respectively. **(B)** Cryo-EM map of the structure colored by the estimated local resolution. **(C)** The model fit within the cryo-EM map. **(D)** Overall interface between mAb #46 and COBRA BC2. The antibody CDR loops and HA structural motifs are labeled. **(E)** Molecular contacts between antibody #46 and COBRA BC2. **(F)** Comparison between how mAb #46 and mAb 1272 bind HA. **(G)** Peptide scanning of mAb #46 against an overlapping library of 13- or 15-mer peptides spanning the ectodomain of the Brisb/08 HA. Peptides covering the HA1 domain are depicted in black and those spanning the HA2 domain are depicted in light blue. Peptide 277–291 showing a statistically significant binding value (*p* < 0.0001) is colored in red.

To gain more insight into the details underlying the broadly neutralizing activity of #46, we solved the structure of the Fab fragment of this antibody in complex with the IBV COBRA HA BC2 (**Figure 6**; **Supplementary Figure 6**). This revealed that mAb #46 binds to an epitope in the head domain that is contained within a single protomer, consistent with the binding data showing reactivity towards an HA monomer (**Figures 4A, B**). Analysis of the model built into the map shows an interface that is formed predominantly between the mAb HC, HA receptor binding site (RBS), and the HA 150-loop. A close examination of the model unveils a mixture of key hydrophobic and electrostatic contributions to the binding interface (**Figures 6D, E**). HC residues I30, F53, and L54 in CDR loops 1 and 2 hydrophobically pack against residues L240 (100% conserved across different Victoria and Yamagata IBV strains), P241 (degree of conservation: 93.1%), and P144 (degree of conservation: 89.7%) in BC2 (**Supplementary Figure 7**). This is complemented by hydrogen bond interactions between the backbone of HC residue L54 with the side chain of N148 (degree of conservation: 65.5%) in BC2, along with the side chain of R56 with N150 (degree of conservation: 55.2%) and the backbone of N148. CDR loop 3 of the HC delves into the RBS, forming extensive contacts in the surrounding pocket. V100c packs into BC2 residues W158 (degree of conservation: 100%), V160 (degree of conservation: 96.6%), and L204 (degree of conservation: 100%, data not shown) in the heart of the pocket, while residues W99 and P100 reside near L240. N100a forms a central, coordinated hydrogen bond interaction with Q242 (degree of conservation: 100%), while backbone-to-backbone hydrogen bonds are formed by P100 and D100b with G141 (degree of conservation: 100%) and T139 (degree of conservation: 96.6%), respectively. Peripherally, the LC of mAb #46 fills out these interactions via a salt bridge between K72 and D164 (degree of conservation: 62.1%) and a hydrogen bond between N57 and the backbone of N163 (degree of conservation: 27.6%).

To gauge how mAb #46 antibody compares to other RBS antibodies, we compared the single-particle cryo-EM structure with an x-ray crystal structure of mAb 1272 bound to an IBV HA (**Figure 6F**) (53). This revealed distinct modes of binding between the two. While both rely primarily on the HC, the orientation of the antibody HC/LC is rotated relative to each other. Within the RBS binding pocket, however, both mediate binding through mimicry of sialic acid through a combination of hydrophobic and electrostatic interactions. In mAb #46, this occurs via Val100c and N100a, while mAb 1272 has a WD motif at residues 103 and 104.

Brisb/08 HA peptide scanning revealed a significant higher binding of mAb #46 to the 277-TYQRGILLPQKVWCA-291 peptide (amino acids 262–266 of the mature HA), which is in partial agreement with the above-described data considering the vicinity of this region to the mAb #46 contact amino acids on the tertiary structure of the HA (**Figure 6G**). Thirteen out of 15 amino acids of this peptide are 100% conserved across different Victoria and Yamagata IBV strains (**Supplementary Figure 7**).

### 3.8 mAb #46 prophylactic and therapeutic protection from lethal IBV challenge in mice

**Figure 7:**
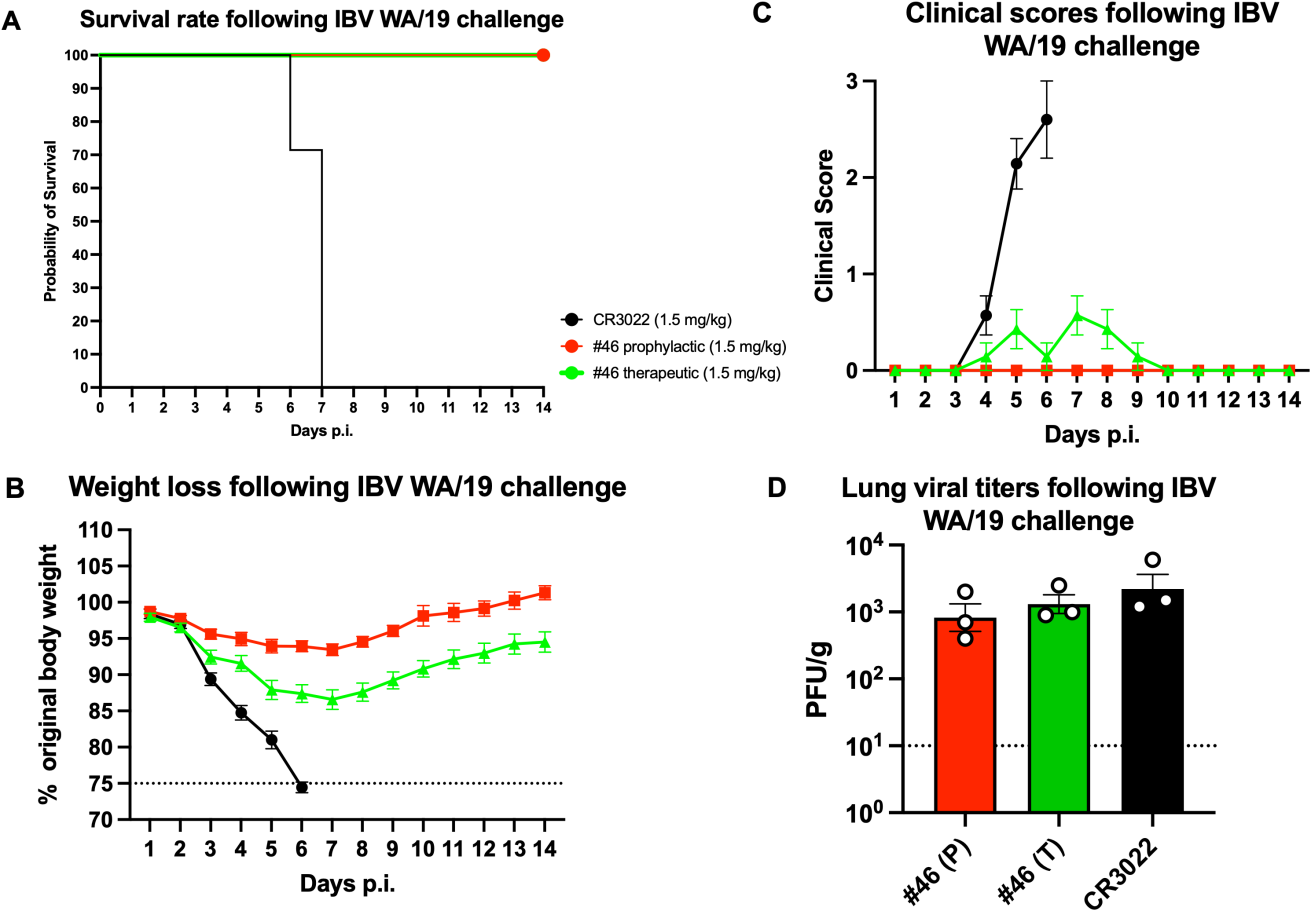
*In vivo* prophylactic and therapeutic IBV challenge studies using passively administered mAb #46. Survival rate **(A)**, weight loss curves **(B)**, and clinical sign scores **(C)** of IBV challenged mice (*n* = 7/group) prophylactically or therapeutically administered with mAb #46. **(D)** Lung viral titers as determined by plaque assay of IBV challenged mice (*n* = 3/group) prophylactically [P] or therapeutically [T] administered with mAb #46. Results of lung viral titers represent one independent experiment performed in triplicate.

To assess whether a mAb endowed with a broad spectrum of HA recognition and a potent neutralizing activity against IBV strains, like mAb #46, was able to protect from an influenza virus challenge, *in vivo* prophylactic and therapeutic studies were conducted in mice. Mice prophylactically or therapeutically administered with 1.5 mg/kg of mAb #46 showed a 100% survival rate along the entire duration of the study, while mice administered with the isotype control (CR3022) all succumbed by day 7 p.i., with 30% of them on day 6 p.i. (**Figure 7A**).

In terms of weight loss, mice prophylactically administered with 1.5 mg/kg of mAb #46 showed a maximum weight loss of 6% on day 7 and 14% for those therapeutically administered with the same dose of the mAb and peaking on the same day. Mice administered with the isotype control reached the weight loss threshold by day 6, after which all mice succumbed to illness or had to be euthanized due to reaching the clinical endpoint (**Figure 7B**, **Table 4**).

**Table 4:** Summary of statistical significance of weight loss across the different mouse groups at different time points.

Weight loss	D1–2	D3	D4	D5	D6	D7	D8–9	D10	D11	D12	D13	D14
CR3022 vs. #46P	ns	<0.0001	<0.0001	<0.0001	<0.0001	na	na	na	na	na	na	na
CR3022 vs. #46T	ns	0.0394	0.0004	0.0015	<0.0001	na	na	na	na	na	na	na
#46P vs. #46T	ns	0.0320	ns	0.0047	0.0004	0.0008	0.0004	0.0016	0.0037	0.0039	0.0066	0.0018

P, prophylactic administration; T, therapeutic administration. Numerical figures represent *p*-values; ns, not significant; na, data not available.

In terms of clinical scores, mice prophylactically administered with 1.5 mg/kg of mAb #46 showed a clinical score of 0 across the entire duration of the study while those therapeutically administered with the same dose of the mAb showed an average clinical score of 0.5 on days 5 and 7 and reaching a clinical score of 0 by day 10 (**Figure 7C**, **Table 5**).

**Table 5:** Summary of statistical significance of clinical scores across the different mouse groups at different time points.

Clinical scores	D1–3	D4	D4	D5	D6	D7	D8–14
CR3022 vs. #46P	ns	0.0286	<0.0001	<0.0001	<0.0001	na	na
CR3022 vs. #46T	ns	ns	0.0004	<0.0001	<0.0001	na	na
#46P vs. #46T	ns	ns	ns	ns	ns	0.0152	ns

P, prophylactic administration; T, therapeutic administration. Numerical figures represent *p*-values; ns, not significant; na, data not available.

The lung viral titers on day 3 following the IBV infection challenge of mice prophylactically or therapeutically administered with mAb #46 were approximately 9 × 10^2^ and 10^3^ PFU/g, respectively, and 2 × 10^3^ PFU/g for the isotype control (**Figure 7D**) with no statistical difference across these three different groups.

## 4 Discussion

Each year, IBVs contribute substantially to global morbidity and mortality. Because constant antigenic evolution results in vaccine–strain mismatch, existing immunization strategies struggle to provide broad protection. Leveraging mAbs with wide-ranging reactivity offers a promising approach to overcome these limitations and is of considerable interest as diagnostic, immunotherapeutic, and immunoprophylactic tools.

Passive immunization with broadly neutralizing antibodies against the major viral surface glycoprotein HA is one of the approaches that have shown great promise for the treatment and prevention of IAV infection. In this study, the binding and functional activity of polyclonal antibodies from 15 individuals against a panel of IBV rHA and strains representative of the two IBV lineages was characterized. Similar to previous studies from our group and others including a larger cohort of individuals spanning different age groups (16, 17, 54–57), we observed a higher magnitude of antibody binding and HAI activity in young adults compared to middle-aged and elderly individuals. As previously speculated, the lower antibody response observed in the elderly population is likely associated with immunosenescence, which is an age-related decline in mounting or recalling the immune response against infections or following vaccination and associated with an increased overall inflammation, which ultimately affects the quality and magnitude of the immune response. While some or all elderly individuals, depending on the season, received the high-dose formulation of the IIV, this factor does not impact the overall antibody response of this age group across seasons as well as compared to the other age groups (young adults and middle-aged) who all received a standard dose of IIV. Similarly, only four out of six middle-aged individuals received the intradermal IIV formulation in the 2013–2014 season. Also in this case, this difference does not have an impact on the overall antibody response of this age group compared to other seasons when all the individuals of this age group received a regular dose of IIV.

A panel of mAbs, specific to HA IBV, with both cross-reactive and lineage-specific binding was also obtained and characterized. Within this study, four new human mAbs specific to the IBV-HA were identified that showed broad reactivity across the two IBV lineages. In particular, mAbs #3576 and #46 had the broadest cross-reactivity, covering strains spanning over 13 and 20 years, respectively, including, in the case of mAb #46, the most recent IBV strains from the Victoria lineage. Importantly, mAb #3576 is also endowed with ADCC activity, suggesting the additional contribution of Fc-mediated activity as a protection mechanism. The cross-reactivity was also reflected by a broad HAI activity, suggesting the recognition of an epitope within the head domain of the HA. Currently, circulating IBV strains only belong to the Victoria lineage, leading to the decision since 2024 to not include a Yamagata strain in the seasonal influenza vaccine. Similarly, mAbs recognizing IBV strains belonging to the Yamagata lineage are less relevant for their consideration in the clinical practice. However, the broad binding and functional profile of mAbs extended to both lineages, like mAb #46 described in this work, reflects their recognition of HA epitopes resistant to antigenic change, and this recognition could be expected to be maintained over a significant period of time. In this regard, 8 out of the 12 BC2 HA contact amino acids with mAb #46 are >89% (with 4 of them 100%) conserved across different historical and recent IBV strains belonging to the Victoria and Yamagata lineages.

In addition, their functional profile was confirmed by an *in vitro* neutralization assay using the live influenza virus MA/12.

Importantly, mAb #46 was able to completely protect mice, either administered prophylactically or therapeutically, from weight loss, clinical signs, and ultimately mortality upon challenge with the WA/19 IBV strain, suggesting that it might have a potential clinical use in an immunoprophylactic or immunotherapeutic setting. Despite divergences in weight loss, clinical signs, and survival, no statistical difference was observed in lung viral titers among the treatment groups. These results may be attributed to the absence of or low difference observed on day 3 p.i. in weight loss, clinical signs, and survival rate that may also be reflected in the absence of divergences in the lung viral titer at that time point among the treatment groups. Lung viral titer differences could have reached statistical significance at later time points (days 4–5 p.i.) when divergences in weight loss, clinical signs, and survival rate were more pronounced. Alternatively or additionally, other non-neutralization antibody-mediated mechanisms of protection may have contributed to this discrepancy.

To better understand the molecular features that mediate broad neutralization, we used single-particle (SPA) cryo-EM to obtain a structure of antibody #46 bound to the IBV COBRA HA BC2. This revealed that, like many other broadly neutralizing antibodies that target influenza, mAb #46 targets a conserved epitope in the RBS. While sharing the same target, however, mAb #46 is distinct from other IBV-targeting antibodies that have been structurally characterized, pointing to an underlying diversity that permits different antibody lineages to converge on a similar molecular target.

The observed consistency in the breadth and magnitude of binding and functional activity between polyclonal sera and their corresponding mAbs suggests that next-generation vaccines incorporating multiple antigenically distinct epitopes could elicit a broader immune response, as previously observed in the context of IAV (58, 59). Such a strategy may enhance the ability to recognize and neutralize a wider range of influenza strains, including emerging drifted variants (60). This is further supported, especially in the young adult population, by the observation of a *bona fide* PB ASC- and B_mem_-derived antibody reactivity against IBV COBRA HA antigens, suggesting the existence of pre-existing COBRA reactive antibodies in the human BCR repertoire.

In this study, it was possible to isolate high-affinity, cross-reactive mAbs from people vaccinated with the QIV SOC, which can maintain binding and resistance to natural antigenic changes over a significant period of time. We have also highlighted the cross-reactivity of the human polyclonal antibodies and of the mAbs against IBV COBRA HA antigens, suggesting that these antibodies may potentially be recalled upon vaccination with these next-generation influenza vaccines.

Upcoming clinical trials with COBRA HA will help confirm this hypothesis and further shed light on the mechanism(s) of broad protection conferred by COBRA vaccination, especially in the context of influenza virus pre-immunity and across different age groups, including high-risk populations.

A primary limitation of our study is the small sample size used to evaluate polyclonal and mAb responses to IBV. Consequently, our findings should be viewed as exploratory and descriptive rather than as statistically definitive conclusions. However, the observed trend of the biological activity of polyclonal sera from the different age groups is consistent with previous studies including larger cohorts of individuals.

## Data Availability

The datasets presented in this study can be found in online repositories. The names of the repository/repositories and accession number(s) can be found in the article/**Supplementary Material**.
