# Supplementary material for: Human broadly neutralizing influenza B virus antibodies recognizing hemagglutinin computationally optimized broadly reactive antigens
Source: Front Immunol. 2026 Mar 25;17:1747235. doi: 10.3389/fimmu.2026.1747235 (PMC13057379; doi:10.3389/fimmu.2026.1747235)
Supplement: Supplementary file 1 [file SupplementaryFile1.docx]

Supplementary Material

## Supplementary Figures


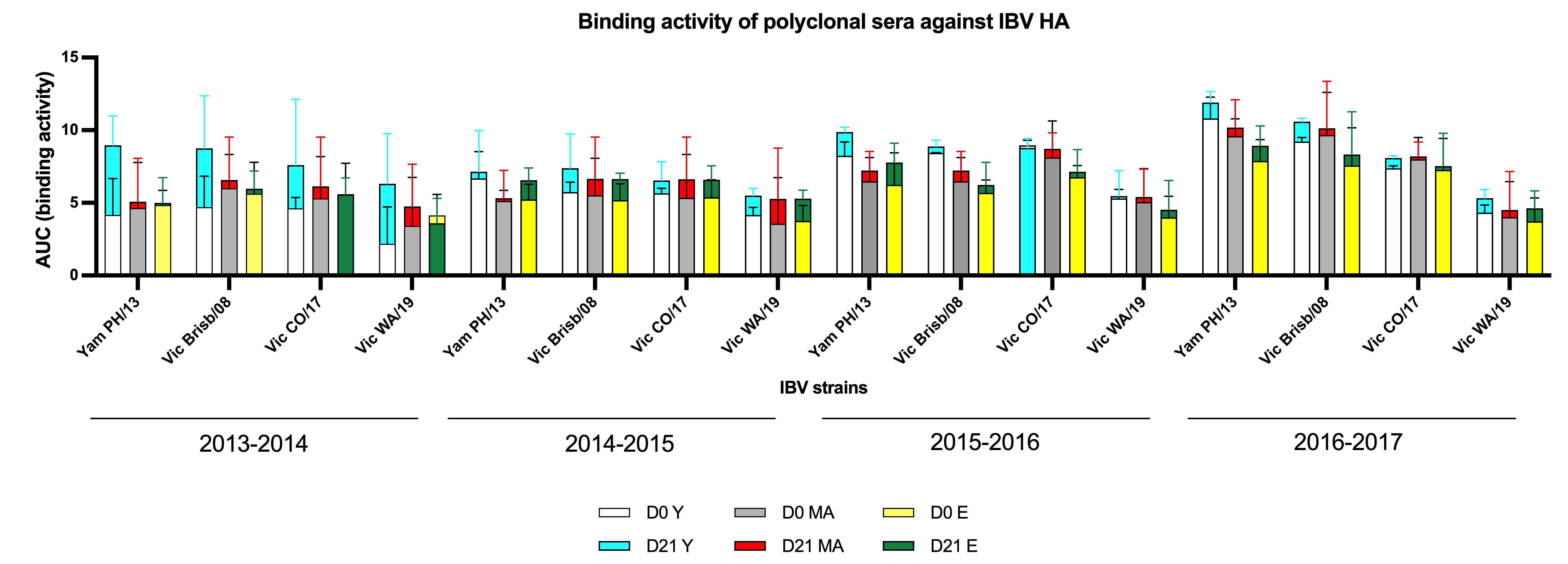


**Supplementary Figure 1.** **Binding activity of polyclonal sera collected from study participants belonging to different age groups.** Breadth and magnitude of binding of young adults (Y), middle-aged (MA) and elderly (E) individuals, at baseline (D0) and 21 days (D21) following administration of QIV over four consecutive influenza seasons. Breadth of binding was determined by ELISA against a panel of rHA representing historical and recent IBV vaccine strains. Binding is expressed as the median plus 95% confidence interval (error bars) of the AUC of the OD values obtained from the serum dilutions of each participant ID against the corresponding IBV HA at D0 and D21 (superimposed bars). ELISA were performed in duplicate for two independent experiments. Results represent one experiment performed in duplicate.


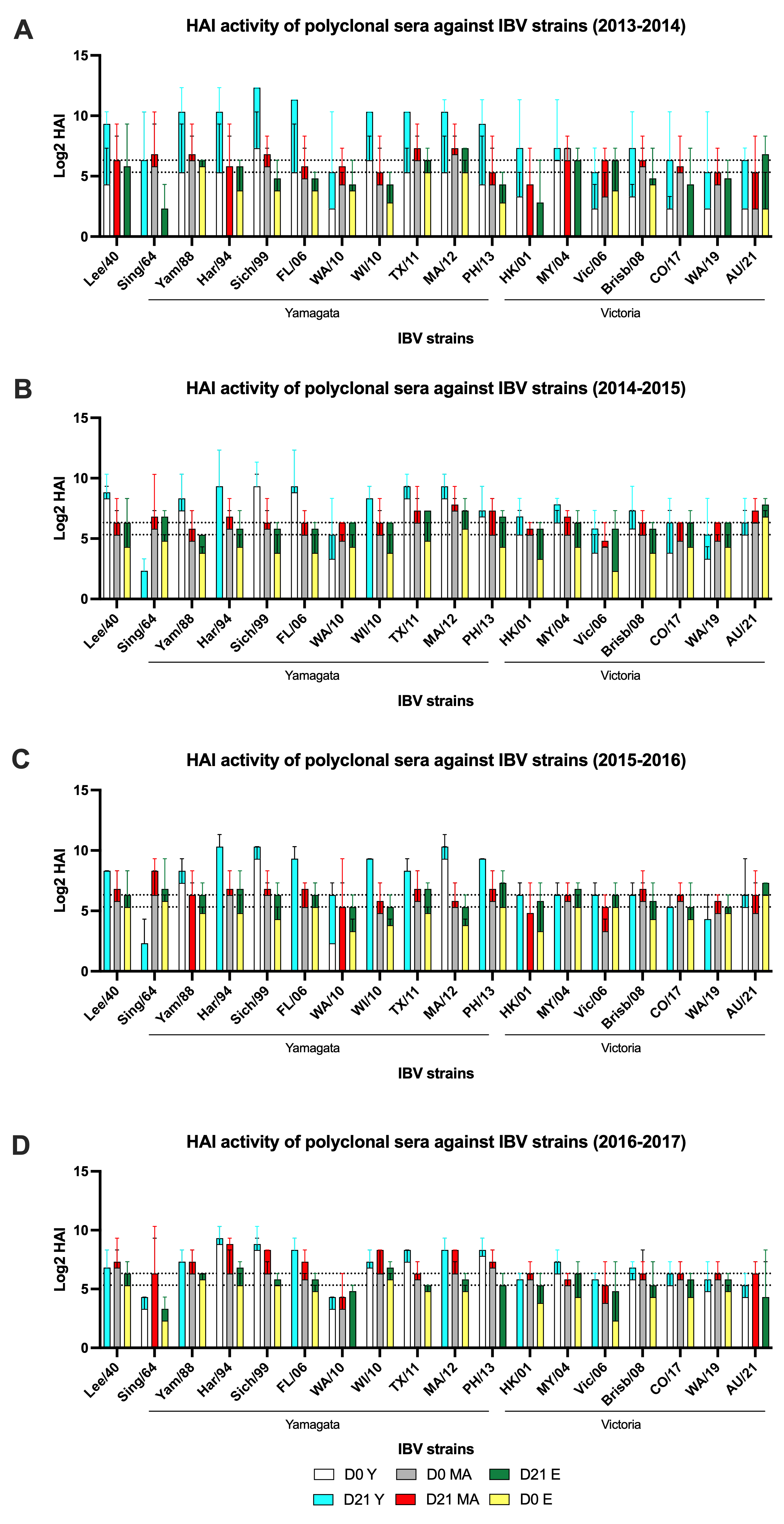


**Supplementary Figure 2.** **HAI activity of polyclonal sera collected from study participants belonging to different age groups.** HAI activity of sera from young adults (Y), middle-aged (MA) and elderly (E) individuals, at baseline (D0) and 21 days (D21) following administration of QIV over four consecutive influenza seasons (**A**-**D**). Breadth of functional activity was determined by HAI assay against a panel of IBV isolates representing historical and recent vaccine strains. HAI is expressed as the median plus 95% confidence interval (error bars) of the Log2 of the reciprocal dilution of the last serum dilution point that inhibited the hemagglutination at D0 and D21 (superimposed bars). Dotted lines on the y axis indicate the 5.32 and 6.32 Log2 values corresponding to the reciprocal of the 1:40 and 1:80 seroprotective threshold antibody titers, respectively. HAI were performed in duplicate for two independent experiments. Results represent one experiment performed in duplicate.

**Supplementary Figure 3.** (**A**) Heatmap of magnitude of IgG, IgM and IgA HA-specific plasmablast (PB)-derived ASCs from PBMCs of study participants belonging to different age groups: young adults (Y), middle-aged (M) and elderly (E) individuals collected 7 days (D7) following QIV administration over four consecutive influenza seasons. HA-specific IgG, IgM and IgA-positive ASCs against PH/13 and BC2 rHA were enumerated and normalized to the number of total IgG, IgM and IgA ASCs. For each independent experiment, PBMCs from the same donor were run in duplicate. Results represent two independent experiments performed in duplicate. (**B**) Heatmap of magnitude of HA-specific IgG secreted from in vitro stimulated PBMCs of study participants collected at baseline (D0) and 21 days (D21) following administration of QIV over four consecutive influenza seasons. Data are presented as a ratio of HA-specific IgG (expressed as μg/mL IgG equivalents) against historical and COBRA rHA and the total IgG (expressed as μg/mL IgG equivalents) secreted from in vitro stimulated PBMCs. For each independent experiment, supernatants from in vitro stimulated PBMCs from the same donor were run in duplicate. Results represent two independent experiments performed in duplicate.

**Supplementary Figure 4.** Magnitude of total (**A**) and HA-specific (**B**) plasmablast (PB)-derived IgG, IgM and IgA ASCs from PBMCs of study participants belonging to different age groups: young adults, middle-aged and elderly individuals collected 7 days (D7) following QIV administration over four consecutive influenza seasons. Spot forming units (SFU) of total and HA-specific IgG, IgM and IgA-positive ASCs against PH/13 and BC2 rHA were enumerated and normalized to 1 million of input cells. For each independent experiment, PBMCs from the same donor were run in duplicate. Results represent two independent experiments performed in duplicate and are expressed as mean plus the standard error of the mean. **p*<0.05; ***p*<0.01; *****p*<0.0001.

**

**Supplementary Figure 5.** Breadth and magnitude of total and HA-specific IgG secreted from in vitro stimulated PBMCs of study participants collected at baseline (D0) and 21 days (D21) following administration of QIV over four consecutive influenza seasons (**A**-**D**). Data are presented as the median and 95% confidence intervals (error bars) of the total and HA-specific IgG equivalents (expressed as μg/mL) against historical and COBRA rHA from in vitro stimulated PBMCs collected at D0 and D21 (superimposed bars). For each independent experiment, supernatants from in vitro stimulated PBMCs from the same donor were run in duplicate. Results represent two independent experiments performed in duplicate.


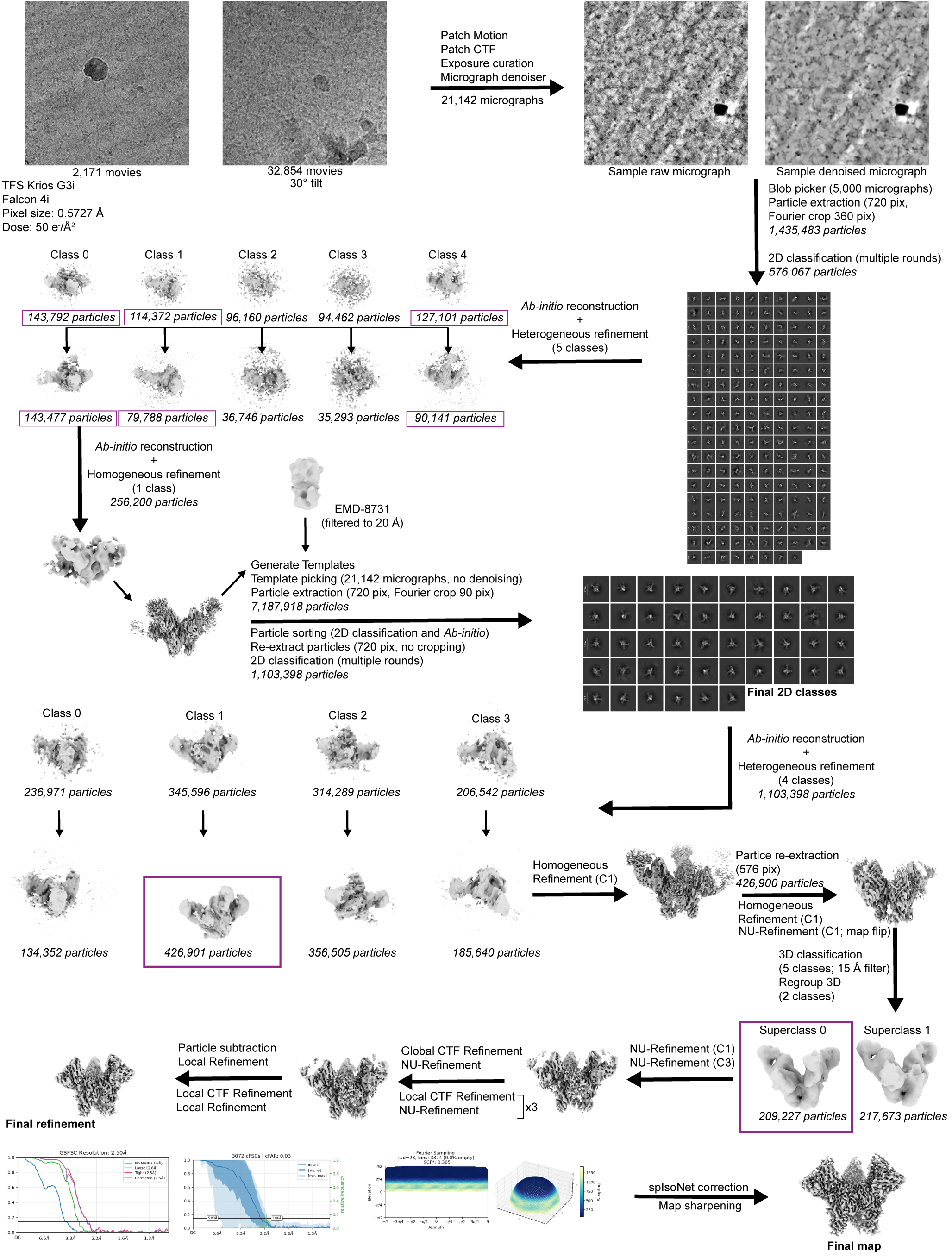


**Supplementary Figure 6.** Single-particle cryo-EM data processing workflow. Overview of the steps that resulted in the final map used for model building. All steps were performed in CryoSPARC v4.5.3 apart from the spIsoNet correction step.

BC1 140_TSGSCPNVTSRSGFFATMAWAVPR-DN_165 238_GGLPQS_243 262_VYQRGVLLPQKVWCA_276

**BC2**  **140_TSGSCPNVTNGNGFFATMAWAVPKNDN_165 238_GGLPQS_243 262_VYQRGILLPQKVWCA_276**

BC3 140_TSGSCPNVTNGNGFFATMAWAVPKNDN_165 238_GGLPQS_243 262_TYQRGILLPQKVWCA_276

BC17 140_TSGSCPNITNGNGFFATMAWAVP---N_165 238_GGLPQS_243 262_VYQRGILLPQKVWCA_276

Lee/40 140_TSGSCPNVANRNGFFNTMAWVIPK-DN_165 238_EGLKQS_243 262_VYQRGVLLPQKVWCA_276

MD/59 140_TSGSCPNVTNGKGFFETMAWAVPK-NK_165 238_EGLKQS_243 262_AYQRGVLLPQKVWCA_276

Vic-HK/72 140_ISGSCPNVTNGNGFFATMAWAVPK---_165 238_EGLPQS_243 262_AYQRGVLLPQKVWCA_276

Vic-Sing/79 140_TSGSCPNVTNGNGFFATMAWAVPK--D_165 238_GGLPQS_243 262_VYQRGVLLPQKVWCA_276

Vic-AA/86 140_TSGSCPNVTNGNGFFATMAWAVPKNNN_165 238_GGLPQS_243 262_TYQRGILLPQKVWCA_276

Vic-Beijing/87 140_TSGSCPNVTNGNGFFATMAWAVPKNDN_165 238_GGLPQS_243 262_TYQRGILLPQKVWCA_276

Vic-Vic/87 140_TSGSCPNVTNGNGFFATMAWAVPKNDN_165 238_GGLPQS_243 262_TYQRGILLPQKVWCA_276

Vic-HK/01 140_TSGSCPNVTNGNGFFATMAWAVPKNEN_165 238_GGLPQS_243 262_TYQRGILLPQKVWCA_276

Vic-MY/04 140_TSGSCPNVTNGNGFFATMAWAVPKNDN_165 238_GGLPQS_243 262_TYQRGILLPQKVWCA_276

*Vic-Brisb/08* 140_TSGSCPNITNGNGFFATMAWAVPKNDK_165 238_GGLPQS_243 *262_TYQRGILLPQKVWCA_276*

Vic-CO/17 140_TSGSCPNITNGNGFFATMAWAVP--DK_165 238_GGLPQS_243 262_TYQRGILLPQKVWCA_276

Vic-WA/19 140_TSGSCPNITNGNGFFATMAWAVP---K_165 238_GGLPQS_243 262_TYQRGILLPQKVWCA_276

Vic-AU/21 140_TSGSCLNITNGKGFFATMAWAVP---K_165 238_GGLPQS_243 262_TYQRGILLPQKVWCA_276

Vic-MD/25 140_TSGSCLNITNGKGFFATMAWAVP---K_165 238_GGLPQS_243 262_TYQRGILLPQKVWCA_276

Vic-MT/25 140_TSGSCLNITNGKGFFATMAWAVPK---_165 238_GGLPQS_243 262_TYQRGILLPQKVWCA_276

Yam-HK/73 140_TSGSCPNVTNGNGFFATMAWAVPK---_165 238_EGLPQS_243 262_AYQRGVLLPQKVWCA_276

Yam-Yam/88 140_TSGSCPNVTSRNGFFATMAWAVPR-D-_165 238_GGLPQS_243 262_VYQRGVLLPQKVWCA_276

Yam-Pan/90 140_TSGSCPNVTSRDGFFATMAWAVPR-D-_165 238_GGLPQS_243 262_VYQRGVLLPQKVWCA_276

Yam-Har/94 140_TSGSCPNATSRSGFFATMAWAVPR-DD_165 238_GGLPQS_243 262_VYQRGVLLPQKVWCA_276

Yam-Jilin/03 140_TSGSCPNATSKSGFFATMAWAVPK-DN_165 238_GGLPQS_243 262_VYQRGVLLPQKVWCA_276

Yam-FL/06 140_TSGSCPNATSKSGFFATMAWAVPK-DN_165 238_GGLPQS_243 262_VYQRGVLLPQKVWCA_276

Yam-WI/10 140_TSGSCPNATSKIGFFATMAWAVPK-DN_165 238_GGLPQS_243 262_VYQRGVLLPQKVWCA_276

Yam-MA/12 140_TSGSCPNATSKSGFFATMAWAVPK-DN_165 238_GGLPQS_243 262_VYQRGVLLPQKVWCA_276

Yam-PHU/13 140_TSGSCPNATSKIGFFATMAWAVPK-DN_165 238_GGLPQS_243 262_VYQRGVLLPQKVWCA_276

Yam-UT/14 140_TSGSCPNATSKIGFFATMAWAVPK-DN_165 238_GGLPQS_243 262_VYQRGVLLPQKVWCA_276

**Supplementary Figure 7.** Alignment of HA sequence fragments (amino acids 140-165 and 238-243) derived from different IBV strains belonging to the COBRA, pre-split, Victoria (Vic) and Yamagata (Yam) lineages containing contact amino acids with heavy and light chains of mAb #46 based on the single-particle cryo-EM BC2 HA-mAb complex structural studies. Conserved HA amino acids interacting with the heavy chain of mAb #46 are highlighted in dark green, while conserved HA amino acids interacting with the light chain of mAb #46 are highlighted in cyan. Amino acids 262-276 are derived from the sequence of the HA peptide (from Vic-Brisb/08, in italics) that showed a higher degree of binding in the HA peptide scanning of mAb #46 where conserved HA amino acids are highlighted in grey. Abbreviations of additional IBV strains included in the alignment and not previously spelled out in the manuscript, pre-split strains: B/Maryland/1959 (MD/59); Victoria (Vic) lineage strains: B/Hong Kong/05/1972 (HK/72), B/Singapore/222/1979 (Sing/79), B/Ann Arbor/1/1986 (AA/86), B/Beijing/1/1987 (Beijing/87), B/Victoria/2/1987 (Vic/87), B/Maryland/01/2025 (MD/25), B/Montana/06/2025 (MT/25); Yamagata (Yam) lineage strains: B/Hong Kong/8/1973 (HK/73), B/Panama/45/1990 (Pan/90) and B/Utah/09/2014 (UT/14). Included are also the COBRA HA sequences for BC1, BC2 (in bold), BC3 and BC17.

## Supplementary Tables

|  | **ELISA** | **2013-2014** | | **2014-2015** | | **2015-2016** | | **2016-2017** | |
| --- | --- | --- | --- | --- | --- | --- | --- | --- | --- |
|  |  | **D0** | **D21** | **D0** | **D21** | **D0** | **D21** | **D0** | **D21** |
| **Yamagata** | **PH/13** | ns | Y-MA**  Y-E** | ns | ns | ns | Y-MA* | ns | ns |
| **Victoria** | **Brisb/08** | ns | Y-MA**  Y-E** | ns | ns | Y-E* | ns | ns | ns |
|  | **CO/17** | ns | Y-E* | ns | ns | ns | ns | ns | ns |
|  | **WA/19** | ns | Y-E* | ns | ns | ns | ns | ns | ns |

**Supplementary Table 1.** **Summary of statistical significance of differences in the binding activity of polyclonal sera collected from study participants belonging to different age groups.** Statistical significance of binding activity (by ELISA) across participants belonging to young adults (Y), middle-aged (MA) and elderly (E) individuals, at baseline (D0) and 21 days (D21) following administration of QIV. The statistical significance here summarized refers to the data represented in Figure 2 and Supplementary Figure 1. **p*<0.05; ***p*<0.01; ns=not significant.

|  | **HAI** | **2013-2014** | | **2014-2015** | | **2015-2016** | | **2016-2017** | |
| --- | --- | --- | --- | --- | --- | --- | --- | --- | --- |
|  |  | **D0** | **D21** | **D0** | **D21** | **D0** | **D21** | **D0** | **D21** |
|  | **Lee/40** | ns | ns | Y-MA**  Y-E** | Y-MA* | ns | ns | ns | ns |
|  | **Sing/64** | Y-E***  MA-E*** | Y-E****  MA-E**** | Y-MA***  Y-E* | Y-MA****  Y-E**** | Y-MA**  Y-E* | Y-MA****  Y-E**** | Y-MA**  MA-E**** | Y-MA*  MA-E**** |
| **Yamagata** | **Yam/88** | ns | Y-MA*  Y-E** | Y-MA**  Y-E*** | Y-MA**  Y-E** | Y-E** | ns | ns | ns |
|  | **Har/94** | ns | Y-MA****  Y-E*** | Y-MA***  Y-E**** | Y-MA**  Y-E*** | Y-MA**  Y-E**** | Y-MA**  Y-E* | Y-E** | Y-MA*  MA-E* |
|  | **Sich/99** | Y-E* | Y-MA****  Y-E**** | Y-MA***  Y-E**** | Y-MA**  Y-E*** | Y-MA**  Y-E**** | Y-MA*  Y-E** | Y-E** | Y-MA**  MA-E* |
|  | **FL/06** | ns | Y-MA****  Y-E**** | Y-MA***  Y-E**** | Y-MA***  Y-E*** | Y-MA***  Y-E*** | Y-MA*  Y-E* | Y-MA*  Y-E** | Y-E* |
|  | **WA/10** | Y-MA* | ns | ns | ns | Y-MA* | ns | ns | ns |
|  | **WI/10** | Y-E** | Y-MA****  Y-E**** | Y-MA**  Y-E**** | Y-E* | Y-MA****  Y-E**** | Y-MA**  Y-E** | ns | ns |
|  | **TX/11** | ns | Y-MA*  Y-E** | Y-E*** | ns | Y-E** | ns | ns | Y-E** |
|  | **MA/12** | ns | Y-MA**  Y-E** | Y-E* | ns | Y-MA***  Y-E**** | Y-MA***  Y-E*** | Y-E** | Y-MA*  MA-E* |
|  | **PH/13** | ns | Y-MA****  Y-E**** | Y-E* | ns | Y-MA*  Y-E** | ns | Y-E* | Y-E* |
| **Victoria** | **HK/01** | ns | Y-MA**  Y-E**** | ns | ns | Y-E* | ns | ns | ns |
|  | **MY/04** | ns | ns | Y-E* | ns | ns | ns | ns | ns |
|  | **Vic/06** | ns | ns | ns | ns | Y-MA* | ns | ns | ns |
|  | **Brisb/08** | Y-MA** | Y-E* | ns | Y-E* | ns | ns | ns | ns |
|  | **CO/17** | Y-MA** | ns | ns | ns | ns | ns | ns | ns |
|  | **WA/19** | Y-MA* | ns | ns | ns | ns | ns | ns | ns |
|  | **AU/21** | ns | ns | ns | ns | ns | ns | ns | ns |

**Supplementary Table 2.** **Summary of statistical significance of differences in the HAI activity of polyclonal sera collected from study participants belonging to different age groups.** Statistical significance of HAI activity across participants belonging to young adults (Y), middle-aged (MA) and elderly (E) individuals, at baseline (D0) and 21 days (D21) following administration of QIV. The statistical significance here summarized refers to the data represented in Figure 3 and Supplementary Figure 2. **p*<0.05; ***p*<0.01; ****p*<0.001; *****p*<0.0001; ns=not significant.

|  | **B_mem_**  **ELISA** | **2013-2014** | | **2014-2015** | | **2015-2016** | | **2016-2017** | |
| --- | --- | --- | --- | --- | --- | --- | --- | --- | --- |
|  |  | **D0** | **D21** | **D0** | **D21** | **D0** | **D21** | **D0** | **D21** |
| **Total IgG** | | Y-MA**  MA-E**** | MA-E** | ns | Y-MA**  MA-E* | Y-MA** | ns | ns | Y-MA**  Y-E* |
| **Yamagata** | **PH/13** | ns | ns | Y-MA** | ns | MA-E* | ns | ns | ns |
| **Victoria** | **HK/01** | ns | Y-E* | ns | ns | ns | ns | ns |  |
|  | **Brisb/08** | ns | Y-E* | ns | ns | ns | ns | ns |  |
|  | **CO/17** | ns | ns | ns | ns | ns | ns | ns |  |
|  | **WA/19** | ns | ns | ns | ns | ns | ns | ns |  |
|  | **AU/21** | ns | ns | ns | ns | ns | ns | ns |  |
| **COBRA** | **BC2** | ns | ns | ns | ns | ns | ns | ns | ns |
|  | **BC3** | ns | ns | ns | ns | ns | ns | ns | ns |
|  | **BC17** | ns | Y-E** | ns | ns | ns | ns | ns | ns |

**Supplementary Table 3. Summary of statistical significance of differences in the binding activity of B_mem_-derived antibodies from in vitro stimulated PBMCs of study participants belonging to different age groups.** Statistical significance of binding activity across participants belonging to young adults (Y), middle-aged (MA) and elderly (E) individuals, at baseline (D0) and 21 days (D21) following administration of QIV over four consecutive influenza seasons. The statistical significance here summarized refers to the data represented in Supplementary Figure 4. **p*<0.05; ***p*<0.01; *****p*<0.0001; ns=not significant.
